# Association between cognitive function and supplementation with omega-3 PUFAs and other nutrients in ≥ 75 years old patients: A randomized multicenter study

**DOI:** 10.1371/journal.pone.0193568

**Published:** 2018-03-26

**Authors:** Joaquin Baleztena, Miguel Ruiz-Canela, Carmen Sayon-Orea, Maria Pardo, Teresa Añorbe, Jose Ignacio Gost, Carmen Gomez, Belen Ilarregui, Maira Bes-Rastrollo

**Affiliations:** 1 Centro Residencial Clínica Universidad de Navarra, Pamplona, Spain; 2 Department of Preventive Medicine and Public Health, University of Navarra, Pamplona, Spain; 3 Navarra’s Health Research Institute (IDISNA), Pamplona, Spain; 4 Biomedical Research Center Network on Obesity and Nutrition (CIBERobn) Physiopathology of Obesity and Nutrition, Institute of Health Carlos III, Madrid, Spain; 5 Department of Preventive Medicine, Complejo Hospitalario de Navarra, Pamplona, Spain; 6 Grupo Amma, Pamplona, Spain; National Cancer Center, JAPAN

## Abstract

A few studies have assessed the association between omega-3 polyunsaturated fatty acids (n-3 PUFA) and cognitive impairment (CI) in very old adults. The aim of this study was to evaluate the effect of a multinutrient supplementation rich in n-3 PUFA on the cognitive function in an institutionalized ≥75-year-old population without CI or with mild cognitive impairment (MCI). A multicenter placebo-controlled double-blind randomized trial was conducted between 2012 and 2013. Cognitive function was assessed at baseline and after one year using 4 neuropsychological tests. Nutritional status was assessed using Mini Nutritional Assessment (MNA). Interaction between Mini-Mental State Examination (MMSE) score and nutritional status were analyzed using linear regression models. A total of 99 participants were randomized to receive placebo or pills rich in n-3 PUFA. After 1-year follow-up, both groups decreased their MMSE score (-1.18, SD:0. 53 and -0.82, SD:0. 63, p = 0.67 for the control and the intervention group respectively). The memory subscale of the MMSE showed an improvement (+0.26, SD:0.18) in the intervention group against a worsening in the control group (-0.11, SD: 0.14; p = 0.09 for differences between groups). Patients at intervention group with normal nutritional status (MNA ≥24) showed an improvement in the MMSE (+1.03, p = 0.025 for differences between 1-y and baseline measurements) against a worsening in the group with malnutrition (MNA<24) (-0.4, p = 0.886 for differences between 1-y and baseline; p of interaction p = 0.05). Supplementation with n-3 PUFA did not show an improvement in the global cognitive function in institutionalized elderly people without CI or with MCI. They only suggest an apparent improvement in memory loss if previously they were well nourished.

## Introduction

According to the World Health Organization, 47.5 million people worldwide have dementia and 7.7 million new cases are developed every year [1]. Dementia is currently one of the most important causes of disability among elderly and it has a high physical, psychological, social and economic impact on patients, their caregivers, families and society. The lack of effective treatments makes a priority the development of preventive strategies to preserve cognition or at least to delay the progression of cognitive impairment to dementia.

Diet is an important area to explore preventive interventions. There is growing evidence showing that certain nutritional factors could be associated with cognitive impairment (CI) [2,3] and they might play an important role in the prevention of dementia [4–7]. Among these nutritional factors, polyunsaturated fatty acids (PUFAs) and specifically omega-3 (n-3 PUFA), Vitamin E, B vitamins, folates, and Ginkgo biloba might be involved [8–13]. In this context, it has been demonstrated that better cognitive results might be obtained using multinutrient supplementation; this is because the possible synergies between the components, similar as it happens with an adequate diet [14–16]. Previous studies have reported beneficial effects over cognition when patients were supplemented with multinutrients, all of those containing n-3 PUFA as principal component [8–9, 17–20].

Results from epidemiological studies have suggested that diets high in PUFAs and n-3 PUFA, docosahexaenoic acid (DHA) and Eicosapentaenoic acid (EPA), might reduce the risk of CI and Alzheimer disease (AD) [21]. However, contradictory results have been observed in interventional studies [22]. Recent meta-analyses have concluded that n-3 PUFA intake could help to prevent CI in elderly [23–26] while others have found opposite results [27–29]. In addition, scarce intervention studies have assessed whether the n-3 PUFA are beneficial for the prevention of CI in patients aged ≥ 75 years and even never has been explored the possible interaction with their prior nutritional status.

Therefore, the aim of this study was first to identify if a multivitamin supplement including n-3 PUFA (Docosohexanoic Acid (DHA) and Eicosapentaenoic Acid (EPA) produces beneficial effects in the cognition of elderly institutionalized patients without CI or with mild cognitive impairment (MCI). And second, we explored a possible interaction between the supplements and the prior nutritional status.

## Materials and methods

### Study population and design

This was a multicenter, randomized, double-blinded, and placebo-controlled trial, study. Participants were randomized to take either one capsule of multinutrients rich in n-3 PUFA, 3 times a day (350 mg n-3 PUFA/capsule) or placebo (gelatin capsule). Randomization was simple with sequentially numbered containers. Patients and researchers in charged to perform the assessments at baseline and after 1 year were blinded to the intervention, MR-C generated the random allocation sequence and JB, MP, TA, JIG, CG and BI enrolled and assigned participants to intervention.

Inclusion criteria were: 1) age ≥75 years, 2) a score between 1 to 3 on the Global Deterioration Scale (GDS) [30–31]. Exclusion criteria were: 1) Illiterate participants or those patients who do not understand the implementation and evaluation of the applied test, 2) patients with neurological diseases, or other systemic alterations and mental disorders poorly controlled, 3) vision and hearing impairments, 4) negative assessment by the researcher based on the evaluation tests, 5) established diagnosis of dementia, and 6) history of epilepsy, or seizures. Participants who suffered during follow-up a sudden and permanent decrease of the functional performance in the Reisberg scale GDS, attributable to a disease or acute life event were also excluded.

Before each patient was included in the study we checked cognitive status using two scales the MMSE and the fast GDS. To include the patients in the study they have to had a fast GDS ≥ 3 (e.g. patients without dementia or with mild cognitive impairment). After this, patients were evaluated and if they accomplished all the inclusion criteria, and they did not have any exclusion criteria they were asked to sign the informed consent, and if they agree they were included in the study. Recruitment of patients started January 2012, and finished December 2012. All patients were followed-up for 1 year. Therefore, follow-up time finished in December 2013.

All participants were institutionalized at one nursing home group with 3 centers in Navarra (Spain) one in Mutilva and two of them located in Pamplona. Recruitment of patients was done evaluating all the residents of the 3 included centers and including all those who accepted to participate and filled all the inclusion criteria as stated before. The AMMA Navarra Institutional Review Board approved the trial protocol (December 7^th^, 2011). All study participants provided written, informed consent. The trial was registered in ClinicalTrials.gov with the name “Dietary supplement for the prevention of cognitive decline in a very elderly population” and number: NCT01817101.

The registration was due after enrolment of participants started in the study because we were aware of the importance to register our trial during follow-up but not at the beginning of the study, taking into account that several years ago it was not so usual to register a small trial. The authors confirm that all on going and related trials for this drug/intervention are registered.

#### Intervention

The nutritional composition of every capsule of the multinutrient supplement was: DHA 250 mg, EPA 40 mg, vitamin E 5 mg, phosphatidylserine 15 mg, tryptophan 95 mg, vitamin B 12 5 μg, folate 250 μg and ginkgo biloba 60 mg. The product was manufactured by Angelini Pharma.

Subjects were randomized to one capsule, orally, three times a day, for breakfast lunch and dinner, or placebo for 12 months.

#### Cognitive and nutritional assessment

Cognitive status was assessed at baseline and after one year of intervention using several scales: 1) The MMSE, validated test in screening for dementia and one of the most used both clinical practice and research, despite its limitations is considered a test of choice for longitudinal monitoring of patients [32–33]. It was used, the Spanish validated version of the Mini-Mental State Examination (MMSE), the “*Mini Examen Cognoscitivo*”(MEC) [34] that is a tool for screening of dementia. This test evaluates 5 items and the maximum possible score is 30 considering that the patient have CI if the score is <23 points. 2) The Global Deterioration Scale (GDS), that grades the CI in 7 stages from less to more severity of the CI; stage 3 corresponds to MCI [30–31]. 3) The Short Portable Mental State Questionnaire by Pfeiffer (SPMSQ), this 10–item scale assess temporal and space orientation, attention, and recent and past memory [35]. The cut-off of this scale were 4 or more mistakes in those patients with low educational level; 3 or more mistakes in literate patients (at least know how to write and read); and 2 or more mistakes in those who had higher educational level. 4) The Semantic Verbal Fluency Test, this test measures the number of elements of one category (e.g. animals) that the patient could mention in one minute [36]. This test allows to correctly classifying 93% of the patients with dementia versus those without dementia. In this case the cut-off point was10 5) Clock Drawing Test, is quick and easy to administer cognitive screening instrument and it taps into a wide range of cognitive abilities including executive functions [37]. The cut-off point was 6, we considered a positive test if the score was ≤ 6, and negative test if the total score was > 6.

The nutritional status was assessed using the Mini Nutritional Assessment (MNA) test [38]. The MNA has been designed to provide a rapid assessment of nutritional risk in elderly. This test is composed of four parts: anthropometric measurements, global assessments, dietary questionnaire and subjective assessment. The maximum score is 30 points. In our study we classified participants in two groups according the nutritional status: well-nourished ≥ 24 and at risk or undernourished those with a score < 24 points.

### Statistical analysis

We calculated 38 participants randomized to each group to detect a mean difference between the intervention group and the group control in 3 points on the MEC scale [39], referring to the difference of pre and post mean difference in the intervention group versus pre and post mean difference in the control group, (standard deviation = 4.5) with 80% power and 5% significance level. We estimated 10% loss to follow-up at 1 year and therefore the estimated total number of participants was 84 [39].

We used unpaired Student’s t test for continuous variables and Chi squared (χ2) test for categorical variables to compare baseline and 1-year follow-up characteristics between intervention and control groups. To assess differences in 1-yr changes between intervention and control groups, since some of the variables were not distributed normally we calculated p values using also non-parametric Mann-Whitney U test. To assess differences between 1-yr and baseline cognitive status variables we used Wilcoxon test.

Multivariate linear regression models were used to analyze difference in score levels between the intervention and control group. Covariates included in these models were: cognitive stimulation (yes, no); physical activity (yes, no); educational level (3 categories), and occupation (3 categories), automatic dummies for educational level and occupation were created and we used the first category as the reference.

Analyses were also stratified according to the nutritional status using the MNA score. We evaluated the interaction between cognitive level determined by MEC scale and the nutritional status. Adjusted means of MEC memory scale using ANCOVA models were calculated for baseline and 1-year of follow-up in control and intervention groups stratified by nutritional status.

As secondary analysis, we calculated effect size (Cohen’s d) of mean differences of cognitive scales after 1 year of follow-up.

All p values presented are two-tailed; p < 0.05 was considered statistically significant. Analyses were performed using STATA/SE version 12.1 (StataCorp, College Station, TX, USA).

## Results

The results are presented in accordance with the CONSORT statement [40–41].

**Fig 1:**
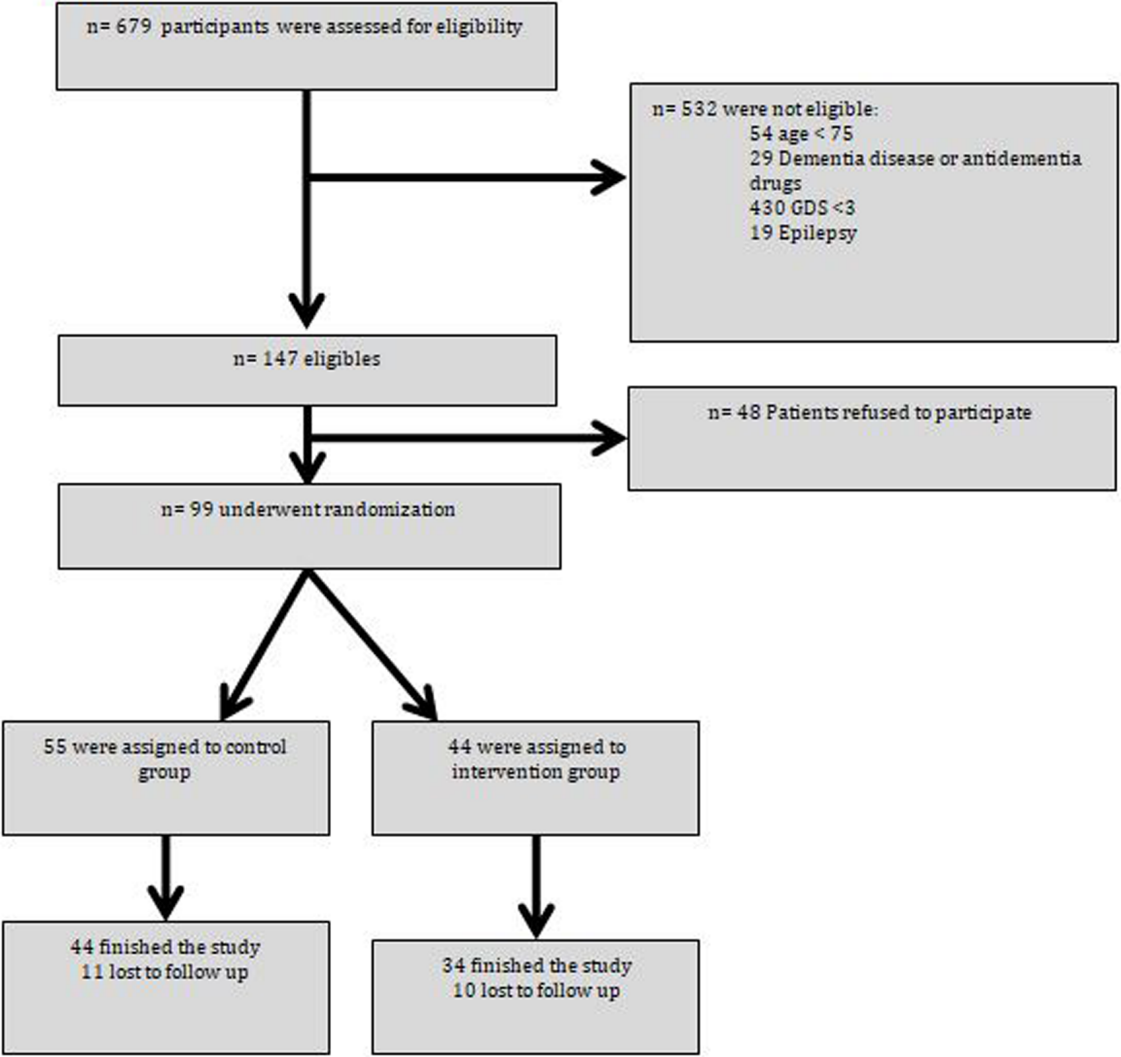
Flow chart of participants.

The flow chart of participants is shown in Fig 1. From 2012 to 2013, 679 institutionalized patients were screened for eligibility and 580 of them were not eligible for the study for the following reasons: 54 were aged < 75 years, 29 had previous diagnosis of dementia or serious cognitive disorder and/or taking antidementia drugs, 430 had GDS greater than 3,19 had history of epilepsy and 48 declined to participate in the study because they did not want to take more pills than the usual medication. A total of 99 patients were randomized either to the intervention or control group. Fifty-five patients were assigned to the control group and 44 to the intervention group. Withdrawals in the study were similar in the intervention and the control group. Reasons for withdrawal were death, left the residential center, and refused to participate during the follow-up of the study.

The baseline characteristics of the participants according to study group are shown in Table 1. Sociodemographic characteristics and morbidity were similar for participants in the both groups.

**Table 1:** Baseline characteristics of participants according to the study group.

	TOTAL	CONTROL	INTERVENTION	p*
	(= 78)	(n = 44)	(n = 34)	
Age (years)	86.9 (5.9)	87.8 (6.5)	85.8 (4.9)	0.127
Men (%)	55 (70.5)	30 (68.2)	25 (73.5)	0.608
CVD (%)	64 (82.1)	35 (79.6)	29 (85.3)	0.512
HT (%)	52 (66.7)	28 (63.6)	24 (70.6)	0.518
Diabetes (%)	16 (20.5)	7 (15.9)	9 (26.5)	0.252
Dyslipidemia (%)	30 (38.5)	18 (40.9)	12 (35.3)	0.613
Stroke (%)	22 (28.2)	12 (27.3)	10 (29.4)	0.835
Thyroid disorders (%)	16 (20.5)	7 (15.9)	9 (26.5)	0.252
Anxiety (%)	17 (21.8)	10 (22.7)	7 (20.6)	0.820
Other diseases (%)	76 (98.7)	42 (97.7)	34 (100)	0.371
Smoking				0.731
Former (%)	15 (19.2)	8 (18.2)	7 (20.6)	
Current (%)	4 (5.1)	3 (6.8)	1 (2.9)	
Alcohol consumption (%)	16 (20.5)	10 (22.7)	6 (17.7)	0.582
Cognitive stimulation (%)	33 (42.3)	23 (52.3)	10 (29.4)	0.043
Physical activity (%)	11 (14.1)	10 (22.7)	1 (2.94)	0.013
Supplements (%)	49 (62.8)	27 (61.4)	22 (64.7)	0.762
Educational level				0.012
Less than primary	14 (17.9)	5 (11.3)	9 (26.5)	
Primary (%)	45 (57.7)	23 (52.3)	22 (64.7)	
Secondary (%)	19 (24.4)	16 (36.4)	3 (8.82)	
Occupation (%)				0.029
Housewife (%)	16 (20.5)	8 (18.2)	8 (23.5)	
Worker (%)	41 (52.7)	19 (43.2)	22 (64.7)	
Professional (%)	21 (26.9)	17 (38.6)	4 (11.8)	
Nutritional status Normal (MNA> = 24) (%)	22 (28.2)	11 (25.0)	11 (32.4)	0.474
Barthel scale> = 60 (%)	49 (62.8)	28 (63.6)	31 (61.8)	0.865

Continuous variables are expressed as means (SD). Categorical variables are expressed as number (%). CVD. Cardiovascular disease; HT. Hypertension; MNA. Mini-Nutritional Assessment

*Unpaired Student’s t test for continuous variable and Chi squared (χ2) test for categorical variables were used to compare baseline characteristics between intervention and control groups

Table 2 shows no differences between control and intervention groups on the cognitive baseline characteristics and also after 1 year of follow-up for the study groups.

There were no adverse effects reported in any of the study groups, and the compliance was 100% in both groups. None of the participants reported to change their usual routine during the study. In nursing homes the regime of life is very stable (diet, time of meals, sleeping hours, and physical activity) this allowed to ensure the compliance of the treatment and also avoid changes in food intake or other type of changes.

**Table 2:** Baseline and 1 year cognitive characteristics of the participants according to the study group.

	TOTAL (n = 78)	CONTROL (n = 44)	INTERVENTION (n = 34)	p*
Pfeiffer baseline	2.23 (1.87)	2.25 (1.88)	2.21 (1.87)	0.918
Pfeiffer 1 year	2.50 (2.02)	2.65 (2.08)	2.29 (1.96)	0.433
MEC total baseline	24.32 (4.29)	24.18 (4.61)	24.50 (3.89)	0.748
MEC total 1 yr	23.29 (5.27)	23.00 (5.54)	23.67 (4.95)	0.577
MEC Orientation baseline	8.51 (1.81)	8.61 (1.88)	8.38 (1.72)	0.579
MEC Orientation 1 yr	8.29 (2.23)	8.23 (2.26)	8.38 (2.22)	0.762
MEC Fixation baseline	3.06 (0.69)	2.98 (0.15)	3.18 (1.03)	0.208
MEC Fixation 1 yr	2.96 (0.19)	2.95 (0.21)	2.97 (0.19)	0.719
MEC Concentration baseline	3.76 (1.60)	3.73 (1.53)	3.79 (1.70)	0.856
MEC Concentration 1 yr	3.40 (1.77)	3.50 (1.69)	3.26 (1.88)	0.563
MEC Memory baseline	1.33 (1.10)	1.32 (1.03)	1.35 (1.20)	0.891
MEC Memory 1 yr	1.38 (1.14)	1.20 (1.07)	1.62 (1.21)	0.114
MEC Language baseline	7.62 (1.45)	7.52 (1.61)	7.74 (1.24)	0.525
MEC Language 1 yr	7.26 (1.81	7.11 (1.94)	7.44 (1.63)	0.432
GDS baseline	2.10 (0.85)	2.07 (0.85)	2.15 (0.86)	0.686
GDS 1 yr	2.24 (0.96)	2.23 (1.08)	2.26 (0.96)	0.873
Verbal fluency baseline	9.85 (4.64)	9.59 (4.77)	10.18 (4.50)	0.583
Verbal fluency 1 yr	9.62 (4.93)	9.44 (5.28)	9.85 (4.52)	0.719
Clock Test baseline	6.13 (3.45)	5.75 (3.37)	6.62 (3.53)	0.274
Clock Test 1 yr	5.86 (3.40)	5.77 (3.07)	5.97 (3.82)	0.800

Values are presented as means (SD)

*Unpaired t test was used to test differences between groups.

### Cognitive results

After 12 months of intervention the cognitive status showed no significant improvement in any of the study groups (Table 3). All participants decreased the score in all the scales, except in the clock-drawing test, which showed an improvement in the control group. However, no differences between groups were statistically significant. When we evaluated each subscale of the MEC scale, the memory subscale showed an improvement in the intervention group in comparison with a decreased in the control group, although differences were not statistically significant (mean difference: +0.27 for the intervention group and -0.11 in the control group; p = 0.09 for differences between groups).

**Table 3:** Mean differences of cognitive scales after 1 year of follow-up between control and intervention group and stratified by nutritional status.

	CONTROL (n = 44)	INTERVENTION (n = 34)	DIFFERENCES BETWEEN CONTROL AND INTERVENTION (95% CI)	p^1^	p^2^
Pfeiffer	+0.41 (1.48)	+0.09 (1.44)	+0.32 (-0.34 to +0.99)	0.341	0.541
*At risk of undernutrition*	+0.42 (1.56)	+0.39 (1.47)	+0.03 (-0.80 to +0.86)	0.936	0.774
*Well-nourished*	+0.36 (1.29)	-0.55 (1.21)	+0.91 (-0.20 to +2.02)	0.104	0.140
MEC total	-1.18 (3.55)	-0.82 (3.69)	-0.36 (-2.00 to +1.28)	0.665	0.867
*At risk of undernutrition*	-1.33 (3.63)	-1.52 (3.76)*	+0.19 (-1.82 to +2.19)	0.851	0.669	
*Well-nourished*	-0.72 (3.41)	+0.63 (3.20)	-1.36 (-4.31 to +1.58)	0.345	0.465	
MEC Orientation	-0.39 (1.65)	0.00 (1.30)	-0.39 (-1.07 to +0.30)	0.265	0.272	
*At risk of undernutrition*	-0.42 (1.78)	-0.13 (1.32)	-0.29 (-1.17 to +0.59)	0.506	0.542	
*Well-nourished*	-0.27 (1.19)	+0.27 (1.27)	-0.55 (-1.64 to +0.55)	0.312	0.282	
MEC Fixation	-0.02 (0.15)	-0.21 (1.04)	+0.18 (-0.13 to +0.50)	0.251	0.406	
*At risk of undernutrition*	-0.03 (0.17)	-0.30 (1.26)	+0.27 (-0.17 to +0.72)	0.221	0.348	
*Well-nourished*	0	0	-	-	-	
MEC Concentration	-0.23 (1.55)	-0.53 (1.81)*	+0.30 (-0.46 to +1.06)	0.431	0.297	
*At risk of undernutrition*	-0.36 (1.27	-0.74 (1.76)*	+0.38 (-0.44 to +1.19)	0.358	0.210	
*Well-nourished*	+0.18 (2.22	-0.09 (1.92)	+0.27 (-1.58 to +2.12)	0.762	0.534	
MEC Memory	-0.11 (0.92)	+0.27 (1.02)	-0.38 (-0.82 to +0.06)	0.090	0.075	
*At risk of undernutrition*	-0.03 (0.88)	+0.04 (0.88)	-0.07 (-0.55 to +0.41)	0.759	0.587	
*Well-nourished*	-0.36 (1.03)	+0.73 (1.19)	**-1.09 (-2.08 to -0.10)**	**0.032**	**0.026**	
MEC Language	-6.32 (1.62)***	-6.11 (1.81)***	-0.20 (-0.98 to +0.58)	0.608	0.442	
*At risk of undernutrition*	-6.24 (1.77)***	-6.04 (1.82)***	-0.20 (-1.17 to +0.78)	0.684	0.417	
*Well-nourished*	-6.54 (1.12)**	-6.27 (1.84)**	-0.27 (-1.63 to +1.09)	0.681	0.837	
GDS	+0.16 (0.78)	+0.12 (0.64)	+0.04 (-0.29 to +0.37)	0.802	0.995	
*At risk of undernutrition*	+0.15 (0.79)	+0.22 (0.73)	-0.07 (-0.49 to +0.35)	0.754	0.476	
*Well-nourished*	+0.18 (0.75)	-0.09 (0.30)	+0.27 (-1.63 to +1.09)	0.277	0.247	
Verbal fluency	-0.37 (3.69)	-0.32 (2.46)	-0.05 (-1.51 to +1.42)	0.948	0.822	
*At risk of undernutrition*	-0.81 (3.44)	-0.61 (2.14)	-0.20 (-1.84 to +1.43)	0.803	0.993	
*Well-nourished*	+0.91 (4.23)	+0.27 (3.04)	+0.64 (-2.64 to +3.91)	0.689	0.947	
Clock Test	+0.02 (2.57)	-0.65 (1.95)	+0.67 (-0.38 to +1.72)	0.210	0.144	
*At risk of undernutrition*	-0.27 (1.96)	-1.04 (2.03)*	+0.77 (-0.31 to +1.85)	0.159	0.162	
*Well-nourished*	+0.91 (3.85)	+0.18 (1.54)	+0.73 (-1.89 to +3.34)	0.567	0.265	

Values are presented as means (SD)

1: p values were calculated using Unpaired t test to assess differences between groups

2: p values were calculated using U Mann-Whitney test to assess differences between groups

*** p <0.001 for differences between baseline and 1-yr follow-up in each group using Wilcoxon test

** p <0.01 for differences between baseline and 1-yr follow-up in each group using Wilcoxon test

*p <0.05 for differences between baseline and 1-yr follow-up in each group using Wilcoxon test

When we calculated effect size Cohen’s d index of mean differences of cognitive scales after 1 year of follow-up results were consistent. All of them presented an effect size small or very small (absolute values from 0.02 to 0.39) (S2 Table).

Similarly, we found none statistically significant differences between groups when we adjusted for potential confounding factors (Table 4).

**Table 4:** Adjusted* mean differences of cognitive scales after 1 year of follow-up between control and intervention group.

Cognitive Scales	CONTROL	INTERVENTION	p value
Pfeiffer	(Ref.)	-0.27 (-1.05 to 0.52)	0.500
MEC total	(Ref.)	0.13 (-1.80 to 2.07)	0.894
MEC Orientation	(Ref.)	0.44 (-0.37 to 1.26)	0.283
MEC Fixation	(Ref.)	-0.30 (-0.67 to 0.07)	0.107
MEC Concentration	(Ref.)	-0.25 (-1.12 to 0.62)	0.565
MEC Memory	(Ref.)	0.32 (-0.19 to 0.83)	0.214
MEC Language	(Ref.)	0.10 (-0.80 to 1.01)	0.823
GDS	(Ref.)	-0.10 (-0.50 to 0.29)	0.614
Verbal fluency	(Ref.)	+0.15 (-1.59 to 1.89)	0.863
Clock Test	(Ref.)	+0.07 (-1.12 to 1.27)	0.904

*Adjusted for cognitive stimulation, physical activity, educational level, and occupation.

### Stratified analysis by nutritional status

**Fig 2:**
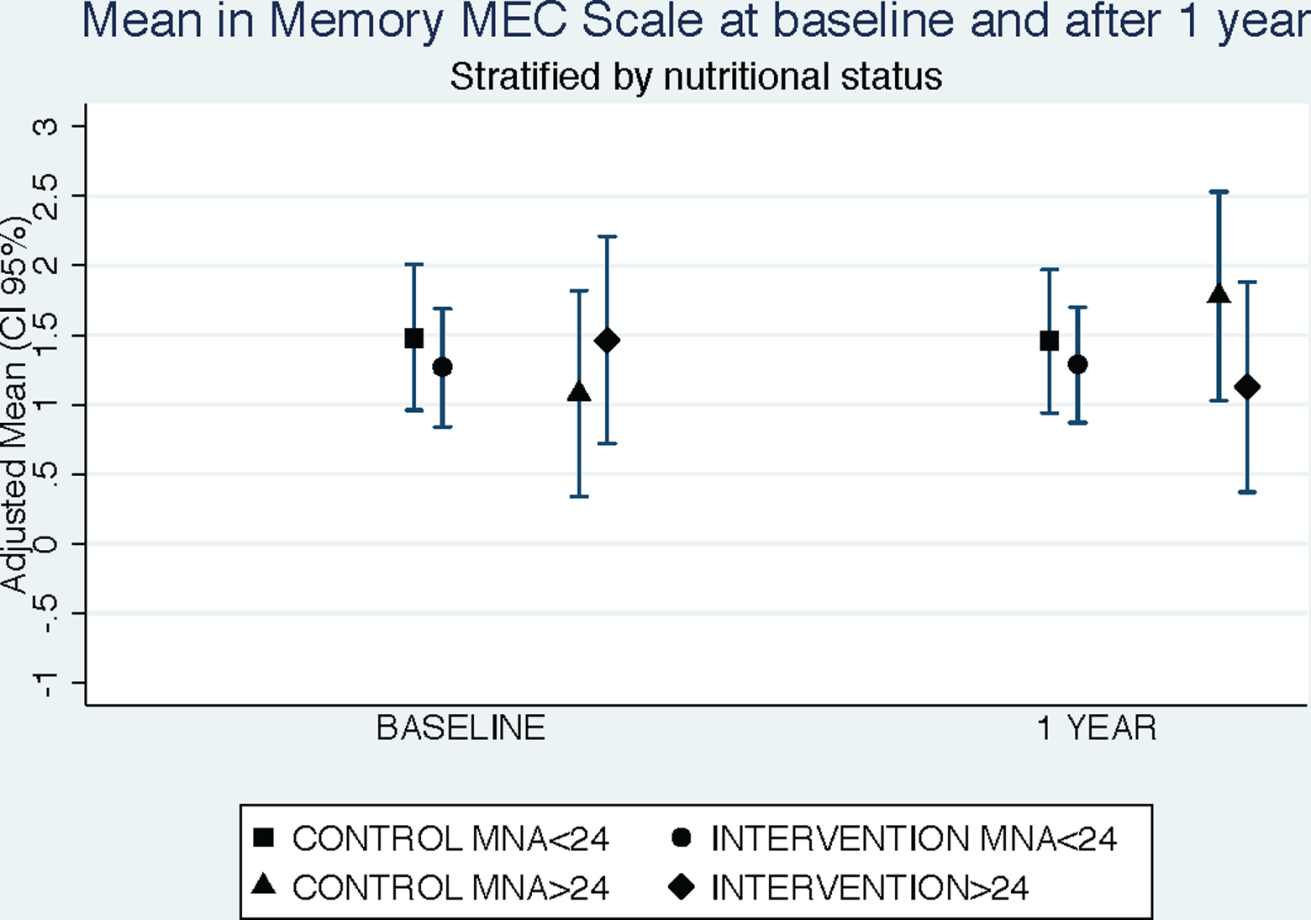
Mean in memory MEC scale at baseline and after 1 year of intervention stratified by treatment and by nutritional status. Adjusted for: cognitive stimulation, physical activity, educational level, and occupation. Ancova test was used to calculate adjusted means.

We stratified participants in 2 groups according to their nutritional status, based on the MNA score, considering those patients with a score ≥24 as well-nourished and those under this score as at risk of under nutrition. We observed a statistically significant improvement in the memory subscale of the MEC scale, in those patients in the intervention group with a MNA ≥24 (Differences between intervention and control group: +1.03 (95% CI: +0.15 to +1.92; p = 0.025 and p for interaction of MNA and intervention on the memory subscale of the MEC = 0.05) (Table 3 and Fig 2).

### Safety, tolerability and compliance

All the participants tolerated well the supplement or placebo without appreciating any side effects. The main complains were the difficulty to swallow the capsules (4 subject in the group intervention and 3 in the placebo) and the excessive number of pills (11 in the group intervention and 14 in the placebo). The compliance of the essay was 100% in both groups.

## Discussion

The results of this study found that a multinutrient supplement, whose main component is n-3 PUFA, does not improve the global cognitive function after 1 year of supplementation. Only, it was observed an apparent reduction in loss of memory in institutionalized patients aged ≥75 years old without DC or with MCI, specially among those previously well-nourished.

Previous studies [21, 42–47] and two recent meta-analyses [25–26] have reported some benefits in memory associated with the intake of n-3 PUFA. Other scales that were used in this study did not showed statistically significant differences between the control group and the intervention group. Overall, this improvement in memory but not in other cognitive function, with multinutrients rich in n-3 PUFA is consistent with other results [26,44], those studies showed beneficial effects in memory performance after supplementation with n-3 PUFA, and no effects were apparent in the cognitive function measured by the MMSE.

The results for the main analysis showed certain favorable effect in the MEC, Pfeiffer, verbal fluency and the GDS of Reisberg in the intervention group, but none of them achieved neither clinical nor statistically significant differences between study groups, this might confirm the conclusions of some studies that did not find beneficial effects in the prevention of the CI with n-3 PUFA supplements [48–52]. Nevertheless, the lack of association in our study might be due to the weak statistical power in our study to detect differences between the control and the intervention group.

Previous studies provided evidence that individuals with established AD do not get any benefit from this type of intervention [53–54] and yet individuals with MCI or AD in incipient stage could benefit from n-3 PUFA supplements [9, 54–56]. On the other hand, the improvements detected are coherent with previous studies [3, 9, 57–62]. Therefore, the evidence still uncertain, especially, when the majority of the intervention studies were conducted on relative small samples. These contradictory findings are shown in the present study in people without AD, showing improvement in memory, but are not conclusive in an improvement of global cognition.

Dietary patterns can better predict the risk of CI than individual nutrients or foods [14]. A recent review proposes to explore potential synergies between nutrients similar to those found on a balanced diet, in this way, better results could be obtained with multinutrient supplementation due to this synergies [15]. A recent study concluded that multinutrient supplement including n-3 PUFA, DHA and EPA, improved the cognition in older women [19]. This study used a multinutrient supplement with a very similar composition of the one we used in our study, finding benefits in the psychomotor speed and verbal memory.

In our study we found that those in the intervention group and well-nourished showed a significant improvement in memory. However, this improvement was not apparent in those who had worse nutritional status. This might be explained because the possibility of better nutrient synergies between the supplement and a good nutritional status. Moreover, malnutrition in elderly is associated with worse health, a decrease in the functional and physiological reserve and fragility, these might conduct to a worse reaction to adverse factors or diseases including cognitive function [63–64].

Only few intervention studies have included patients older than 65 years [18, 20, 48, 51–53]. For example, one study conducted in patients with a mean age of 81.1 years found a slight improvement in career’s visual analogue rating (p = 0.02) and concluded that it was unlikely to find any clinically important treatment effects of EPA on cognition during 12 weeks of treatment period [53]. Other study conducted on patients with a mean age of 76 years and with mild to moderate AD, concluded that supplementation with DHA compared to placebo did not diminish the rate of cognitive and functional deterioration in patients with mild to moderate AD [51]. In the same line, an intervention over 25 patients (mean age of 86 years) found, that a dietary supplement based on an oily emulsion of DHA-phospholipids containing tryptophan and melatonin showed a significant treatment effect for the MMSE, a positive trend for the semantic verbal fluency, and the olfactory sensitivity assessment, and as regards the nutrition evaluation, the supplemented group showed an improvement in the MNA in comparison with the control group [20]. Or another intervention study over 50 patients aged 65 years with MCI found that compared with the Linoleic acid supplemented group the DHA group improved in verbal fluency (Initial Letter Fluency) (p = 0.04), and there were no treatment effects on other cognitive assessments [57].

Two bigger studies found that higher adherence to supplementation intervention was associated with lower AD incidence [18], and the other found that there was no change in cognitive function scores over 24 months [48].

In general, using a multinutrient supplements limits the possibility to attribute only to one of the components all the beneficial effects, even though, the principal component was the n-3 PUFA, however this fact allows to explore potential synergies between nutrients that, can mimic what it is found on a balanced diet [14–16]. It is very difficult to evaluate the association between each individual nutrient and the risk of dementia, because everybody eats complex combinations of nutrients that might have synergic effects through non well known mechanisms [65]. A recent systematic review that analyzed the association between supplements and dementia found that studies conducted with multinutrients supplementation had better results over dementia [66].

A limitation of this study was the small simple size, another limitation was the heterogeneity of the population included in our study including patients without memory impairment and patients with MCI. Probably a longer treatment period may be necessary to demonstrate the efficacy of the supplementation. Finally, the only positive finding in memory loss was based on a secondary analysis carried out to test multiple comparisons. We cannot rule out the existence of this result by chance. Some of the strengths of our study can be also considered as limitations, for example the type of studied population, in our study we included older patients institutionalized in a nursing home, this allowed to ensure the compliance of the treatment and also avoid changes in food intake or other type of changes because its regime of life is very stable (diet, time of meals, sleeping hours, and physical activity) but this can be consider as a limitation in our study in order to extrapolate our results to general population that lives in the community. On the other hand, using a multinutrient complex as intervention treatment might cause interactions between the different nutrients, and also limit the possibility of attributing the effects to specifically one of them, although the main component was n-3 PUFA.

Due to lack of statistical power, these observations can be considered only as preliminary results. More studies are needed with older people, applying specific tests of memory and with greater number of participants to confirm our results.

## Conclusions

The results of this randomized, double blind, placebo-controlled trial found no improvement in global cognitive function during one year of supplementation with a multinutrient supplement whose main component is the n-3 PUFA. They only suggested an apparent improvement in memory loss in institutionalized elderly people without CI or with MCI if previously they were well nourished. More studies similar to this are needed, to confirm our results.

## Supporting information

S1 Table CONSORT checklist. (DOC)

S2 Table Effect size (Cohens’s d) of mean differences of cognitive scales after 1 year of follow-up. (DOCX)

S1 File Original protocol. (DOC)

S2 File English translated protocol. (DOCX)

S3 File Data set of the project. (DTA)
