# Supplementary material for: Association between cognitive function and supplementation with omega-3 PUFAs and other nutrients in ≥ 75 years old patients: A randomized multicenter study
Source: PLoS One. 2018 Mar 26;13(3):e0193568. doi: 10.1371/journal.pone.0193568 (PMC5868762; doi:10.1371/journal.pone.0193568)
Supplement: S2 Table — (DOCX) [file pone.0193568.s002.docx]

**S2 Table. Effect size (Cohen’s d) of mean differences of cognitive scales after 1 year of follow-up.**

| Cognitive Scales | Cohen’s (95% CI) |
| --- | --- |
| Pfeiffer | 0.22 (-0,23 to 0.67) |
| MEC total | -0.10 (-0.55 to 0.35) |
| MEC Orientation | -0.26 (-0.71 to 0.19) |
| MEC Fixation | -0.39 (-0.19 to 0.71) |
| MEC Concentration | 0.18 (-0.27 to 0.63) |
| MEC Memory | -0.39 (-0.84 to 0.06) |
| MEC Language | -0.12 (-0.56 to 0.33) |
| GDS | 0.06 (-0.39 to 0.50) |
| Verbal fluency | -0.02 (-0.47 to 0.44) |
| Clock Test | 0.29 (-0.16 to 0.74) |
