# Supplementary material for: Association between cognitive function and supplementation with omega-3 PUFAs and other nutrients in ≥ 75 years old patients: A randomized multicenter study
Source: PLoS One. 2018 Mar 26;13(3):e0193568. doi: 10.1371/journal.pone.0193568 (PMC5868762; doi:10.1371/journal.pone.0193568)
Supplement: S1 File — (DOC) [file pone.0193568.s003.doc]

**protocolo de ensayo:**

**COMPLEMENTO ALIMENTICIO Y EVOLUCIÓN**

**DEL DETERIORO COGNITIVO en personas de edad avanzada**

**tIPO DE ESTUDIO: MULTICÉNTRICO, OBSERVACIONAL. LONGITUDINAL, ALEATORIZADO FRENTE A PLACEBO**

Dr. Joaquín Baleztena Gurrea

Centro Gerontológico “AMA ARGARAY”

Pamplona

###### ÍNDICE

1. RESUMEN..……………………………………………………………………………………….. 1
2. JUSTIFICACIÓN DEL ESTUDIO…………………………………………………………… 3
3. UTILIDAD POTENCIAL DEL ESTUDIO…………………………………………………. 7
4. OBJETIVOS……………………………………………………………………………………….. 8
5. DISEÑO DEL ESTUDIO……………………………………………………………………….. 8
6. PLAN DE ANÁLISIS ESTADÍSTICO………………………………………………………. 13
7. ASPECTOS ÉTICOS……………………………………………………………………………. 13
8. ASPECTOS ORGANIZATIVOS…………………………………………………………….. 14
9. INFORME FINAL……………………………………………………………………………….. 15
10. ANEXOS…………………………………………………………………………………………… 16

**RESUMEN**

- 1. **. Tipo de estudio**

Estudio piloto multicéntrico, observacional, longitudinal, aleatorizado frente a grupo control (con placebo).

**1.2. Identificación del promotor y dirección**

Dr. Joaquin Baleztena Gurrea

Gerontólogo

Centro Gerontológico “Amma Argaray”

C/ Valle de Egües 1

21003 PAMPLONA

**1.3. Título del estudio**

Complemento alimenticio y evolución del deterioro cognitivo en ancianos de edad avanzada

1.4. Centros donde se prevé realizar el estudio

Centros de pertenecientes a la Institución: “Amma Navarra”

1. Nombre: Centro Gerontológico Amma Argaray

2. Nombre: Centro Gerontológico Amma Oblatas

3 Nombre: Centro Gerontológico Amma Mutilva

PAMPLONA

**1.5. Investigador-Coordinador del estudio**

Dr. Joaquin Baleztena Gurrea

Gerontólogo

Centro Gerontológico “Amma Argaray”

C/ Valle de Egües 1

21003 PAMPLONA

**1.6. CEIC que evalúa el estudio**

Comité de Ética Asistencial e Investigación Clínica (CEAIC) del Centro Gerontológico “Amma Argaray”

# **1.7. Objetivo principal**

Estudiar si la administración a lo largo de un año de un complemento en la dieta compuesto por ácidos grasos omega-3 y otras sustancias (EPA, fosfatidilserina, ácido fólico, vitamina B12, vitamina E y *Gingko biloba*) contenidas en el “complemento dietético” de referencia (ANEXO 1), produce un mejor rendimiento cognitivo en los sujetos de edad avanzada sin déficit previo o con un déficit cognitivo ligero.

**1.8. Enfermedad o trastorno en estudio**

Pacientes de edad avanzada sin deterioro cognitivo o con deterioro leve.

**1.9. Población de estudio y número total de sujetos**

Sujetos sin deterioro cognitivo o con deterioro leve residentes en tres centros gerontológicos del grupo Amma en Navarra. En este estudio piloto se pretende incluir un total de 170 sujetos (150 + aprox 10%), 85 suplementando su dieta con el producto de referencia y 85 administrándoles placebo.

Crook TH, Bartus RT, Ferris SH, Whitehouse P, Cohen GD, Gershon S. Age Associated Memory Impairment: proposed diagnostic criteria measures of clinical change. Report of a NIMH work group. Developmental Neuropsychology 1986; 2: 261-276.

**Criterios de inclusión**

- Pacientes de edad igual o mayor de 80 años.
- Pacientes sin deterioro cognitivo o con deterioro leve, seleccionados mediante escalas cognitivas teniendo en cuenta el informe de la persona, de sus familiares y el juicio global del examinador. Criterio: Global Deterioration Scale GDS (Escala de deterioro global) de Reisberg estadío menor de 4 (GDS 1,2 y 3)
- Que acepten entrar en el estudio y firmen el consentimiento informado.
- Los sujetos con enfermedades sistémicas se incluirán si se juzga que están controladas y que esta enfermedad no produce durante el estudio alteración de tipo cognitivo en el sujeto. Las alteraciones sensoriales (de la visión y audición) se incluirán si se juzga que no son graves y por lo tanto no comprometen los resultados.

**Criterios de exclusión**

- Nivel cultural que impida la comprensión, realización o valoración de los test aplicados.
- Tener enfermedades neurológicas, u otras alteraciones sistémicas o trastornos mentales mal controlados. Este dato es obtenido por historia clínica.
- Valoración negativa por parte del investigador a la vista de las pruebas habituales previas.
- Diagnóstico de Demencia establecida
- Sujetos que a lo largo del estudio sufran una disminución brusca del rendimiento funcional de estadío en la escala GDS de Reisberg, de forma permanente, atribuible a una enfermedad o acontecimiento vital agudo.
- Sujetos con antecedentes de epilepsia, crisis comiciales o convulsiones

###### JUSTIFICACIÓN DEL ESTUDIO

El Deterioro Cognitivo Leve (DCL) o *Mild Cognitive Impairment* (MCI) es un estado de difícil diagnóstico, que se encuentra entre el paso del envejecimiento normal a la demencia leve e incluye el riesgo de una demencia futura. Se sabe que aproximadamente el 60-65% de las personas con un empeoramiento congnitivo leve desarrollan demencia a lo largo de su vida. Para algunos autores la progresión desde que aparece el DCL hasta que se detecta la aparición de demencia es de unos 18 meses (Busse et al., 2006).

La prevalencia de DCL es elevada en la población anciana y dicho dato depende de varios factores, siendo la escala diagnóstica utilizada uno de los importantes. Una búsqueda bibliográfica ha aportado dos estudios de Bermejo, publicados 1997 (prevalencia: 19-23%) y 2002 (prevalencia 13.8-19,9%) en población española.

En base a lo aportado por Fisher y sus colaboradores (2007) se sabe que el 10-15% de pacientes con DCL evolucionan al año a Enfermedad de Alzheimer frente a sujetos sanos que lo hicieron en un 1-2%. Un estudio reciente valora en un 19% el porcentaje de diagnosticados de DCL que presentan Enfermedad de Alzheimer al cabo de 3 años (Okonkowo et al., 2008).

Por su parte Fisher (2007) habla de un 46% de DCL que degenera en Enf. De Alzheimer a los 30 meses. En línea con lo referenciado, Salloway (2006) da una media de progresión del 16% por año.

Realmente hay una divergencia entre los distintos estudios cuando valoran porcentajes de prevalencia e incidencia de la enfermedad, aspecto que puede vincularse en parte con los test utilizados (Petersen et al., 2001).

El DCL es un síndrome que se manifiesta con déficits cognitivos y nula o mínima interferencia con la actividad diaria, no cumpliendo criterios de demencia. Sin embargo, esta definición actualmente se complementa con criterios fisiológicos, patológicos y presencia o no de déficits cognitivos amnésicos (Frutos-Alegría et al., 2007).

Con el fin de detectar precozmente la demencia y valorar su evolución se han desarrollado y están apareciendo diversos test de medición pero ninguno de ellos se aplica individualmente como herramienta diagnóstica (Boustani et al., 2003).

Habitualmente los test son sensibles al diagnóstico cuando las puntuaciones son bajas mientras que, cuando las puntuaciones son elevadas y cercanas al máximo valor del test la variabilidad puede hacer difícil una precisión diagnóstica. Éste es el caso de diagnosticar el deterioro cognitivo leve.

El MMSE, es sin duda el test más validado y utilizado pero ofrece problemas en cuanto a su fiabilidad según la edad de las personas y su nivel cultural, por ello se planteó una tabla de adecuación que ofrece la posibilidad de puntos de corte (Crum, 1993). Existe la versión validada para la población española con el nombre de *Miniexamen Cognitivo* (MEC) de Lobo (Lobo 1979, 1999).

Se ha hablado de la escasa fiabilidad del MMSE a la hora de detectar estados iniciales de Alzheimer debido principalmente al uso de *ítems* muy simples para valorar la memoria y el lenguaje. Sin embargo, se considera el MMSE como el test de elección para el seguimiento longitudinal de los pacientes (Sarasola et al., 2004).

La valoración de la discapacidad funcional es una parte esencial del proceso de diagnóstico y de la planificación en los pacientes con demencia. Se deben utilizar escalas funcionales diseñadas para la población con deterioro cognitivo. Con frecuencia se usan las que han sido diseñadas para valorar el estado funcional de la población anciana en general. Estas escalas no son las más adecuadas para la valoración de poblaciones con deterioro cognitivo. Las escalas que mejor reflejan los déficits son la CDR (Clinical Dementia Rating) y la GDS (Global Deterioration Scale) de Reisberg. La GDS establece siete estadíos posibles desde normal hasta deterioro muy severo. (Antúnez, 2005)

Volviendo al DCL, cada vez más aparecen trabajos en los que la ingesta de ácidos grasos poliinsaturados omega-3 se vinculan a un menor deterioro cognitivo. El consumo de pescado rico en omega-3 (DHA y EPA) parece disminuir el riesgo de deterioro cognitivo y demencia incidental (Cochrane, 2006), incluso algunos investigadores muestran que el consumo de ácidos grasos poliinsaturados omega-3 puede retrasar o prevenir la enfermedad de Alzheimer.

En este sentido, Morris et al., entre los años 1993 y 2000, con la finalidad de evaluar si la ingesta de diferentes tipos de ácidos grasos omega-3 disminuían el riesgo de desarrollar la enfermedad de Alzheimer, iniciaron un estudio poblacional de modo prospectivo en una población de 815 sujetos de edades comprendidas entre los 65 y los 94 años que no padecían enfermedad de Alzheimer, a los que fueron evaluando a lo largo de un período medio de 3,9 años. Al finalizar el estudio, comprobaron que en el grupo de sujetos que habían consumido ácidos grasos poliinsaturados omega-3 (DHA, EPA y alfa-linoléico) presentaban una reducción del 60 % del riesgo de presentar enfermedad de Alzheimer (Morris et al., 2003).

En el mismo sentido, Freund-Levi et al., en el año 2006, quisieron comprobar si una dieta suplementada con ácidos grasos omega-3 administrada durante un año modificaba el curso evolutivo de la enfermedad de Alzheimer en fases de leve a moderada. Para ello, seleccionaron y siguieron a lo largo de un año una muestra de distribución aleatoria de 174 pacientes diagnosticados de enfermedad de Alzheimer en fases de leve a moderada, los cuales fueron tratados de modo doble ciego, bien con una dieta suplementada con ácidos grasos omega-3 (1,7 gramos de DHA y 0,6 gramos de ácido eicosapentaenoico) o bien con placebo. Sólo un grupo de pacientes recibió placebo durante 6 meses, ya que a partir del sexto mes ambos grupos recibieron el mismo suplemento de ácidos grasos omega-3 durante los otros 6 meses. A los 6 meses se realizó el primer control cognitivo y en él sólo se observaron diferencias significativas en el subgrupo de pacientes con enfermedad de Alzheimer con un déficit cognitivo muy leve (MMSE > 27 puntos), que fueron favorables para aquellos pacientes que habían sido tratados con ácidos grasos omega-3 respecto al grupo placebo. En el resto de los pacientes, no se hallaron diferencias significativas en las puntuaciones globales cognitivas. Cuando todos los enfermos comenzaron a recibir suplementos de ácidos grasos omega-3, entre los 3 y los 6 meses, se observó una interrupción en la progresión del deterioro cognitivo similar para todos ellos.

Kotani et al., 2006 seleccionó 39 pacientes (21 con deterioro cognitivo leve, 10 con lesiones cerebrales orgánicas y 8 con enfermedad de Alzheimer). Se les administró: 240 mg de DHA y 40 mg de ARA. A los 90 días del tratamiento, los pacientes con DCL que fueron tratados presentaron mejorías en la memoria inmediata y la atención. El grupo con lesiones cerebrales orgánicas presentaron mejoría en la memoria tardía e inmediata.

Un estudio más reciente, publicado en Archives of Neurology. En él cual se hacía un seguimiento de 899 sujetos sin demencia, concretamente a una subpoblación del estudio Framingham, durante 9 años. Los resultados mostraron que tras medir la cantidad de DHA en las membranas celulares, se observó que efectivamente la población que estaba en el cuartil superior en la presencia de este ácido graso omega-3 en la membrana tenía una incidencia claramente menor de demencia (Schaefer et al., 2006).

Son varios los estudios disponibles que preconizan el uso de DHA en la prevención de la enfermedad de Alzheimer (Conquer 2000; Tully 2003; Barberger-Gateau 2002; Huang 2005).

Con independencia del interés que supone obtener datos del efecto de los omega-3 en la población española, los estudios publicados comprenden franjas de edad amplias. Aunque sea poco frecuente contemplar deterioros cognitivos iniciales en ancianos de 80 o más años resulta de interés poder valorar en este colectivo el uso de dichos ácidos grasos.

Una ventaja añadida a este diseño de estudio, además de disponer de grupo control, es la posibilidad de disponer de una población bastante homogénea, en especial cara a sesgos tan frecuentes como son las variaciones en la alimentación o el cumplimiento de las recomendaciones diarias de ingesta del complemento alimenticio.

Por lo anteriormente expuesto, se ha planteado este estudio para valorar los efectos sobre las áreas cognitivas de los sujetos de edad avanzada mediante un complemento con ácidos grasos poliinsaturados omega-3 en pacientes con DCL. El compuesto utilizado en este estudio presenta como componente mayoritario DHA 340mg, y EPA 40mg. También pequeñas cantidades de, fosfatidilserina, ácido fólico, vitamina B12, vitamina E y *Gingko biloba*. Este estudio se centra en un compuesto enriquecido principalmente por DHA porque el DHA ha sido asociado en la bibliografía científica con bajo riesgo de desarrollo de enfermedad de Alzheimer.

Para dicho estudio se ha pensado en utilizar cuestionarios de uso común en la valoración de este tipo de pacientes: MEC de Lobo de 35 puntos, Fluencia Verbal, Test del Reloj, SPMSQ de Pfeiffer y GDS de Reisberg.

*BIBLIOGRAFÍA*

Aguado C, Martínez J, Onís MC et al. Adaptación y validación al castellano de la versión abreviada de la “Geriatric Depresión Scale” (GDS) de Yesavage. Atención Primaria, 26, supl 1, 328, 2000.

1. Barberger-Gateau P, Letenneur L, Deschamps V, Pérès K, Dartigues JF, Renaud S. Fish, meat, and risk of dementia: cohort study. BMJ, 325, 7370, 932-3, 2002.
2. Bermejo FP, Gabriel RS, Vega SQ, Morales JM, Rocca WA, Anderson DW. Problems and issues with door to door, two phases surveys: An iIlustration from central Spain. Neuroepidemiology, 20:225-231, 2001.
3. Bermejo FP, Portera A, Gabriel RS et al. The prevalence of dementia and cognitive impairment in three sites in central Spain. A door-to-door Study in the Elderly. Neuroepidemiology, 16:7, 1997.
4. Boustani M, Peterson b, Hanson L, Harris R, Lohr: Screening for dementia in primary care: A summary of the evidence for the U.S. preventive Pervices Task Forse. Annals of Internal Medicine, 138, 927-93, 2003.
5. [Busse A, Angermeyer MC, Riedel-Heller SG.](../../../../../pubmed/17077428%3Fordinalpos=14&itool=EntrezSystem2.PEntrez.Pubmed.Pubmed_ResultsPanel.Pubmed_DefaultReportPanel.Pubmed_RVDocSum) Progression of mild cognitive impairment to dementia: a challenge to current thinking. Br J Psychiatry, 189, 399-404, 2006.
6. Conquer JA, Tierney MC, Zecevic J, Bettger WJ, Fisher RH. Fatty acid analysis of blood plasma of patients with Alzheimer´s disease, other types of dementia. Lipids, 35, 12, 1305-1312, 2000.
7. Crum RM, Anthony J.C, Bassett SS, Folstein MF. Population-based norms for the Mini-Mental State Examination by age and educational level. Journal of the American Medical Associatione 269, 18, 2386-239, 1993.
8. Fisher P, Jungwirth S,Zehetmayer, Weissgram S et al. Conversion from subtypes of mild cognitive impairment to Alzheimer dementia. Neurology, 68, 288-291, 2007.
9. Folstein M, Folstein S, McHugh P. Mini-Mental State. A practical method for grading the cognitive state of patients for the clinical. Journal of Psychiatric Research, 12, 189-198, 1975.
10. Freund-Levi Y, Eriksdotter-Jönhagen M, Cederholm T, Basun H, Faxen-Irving G. Ω-3 Fatty acid Treatment in 174 patients with mild to moderate Alzheimer Disease: Omega AD study. Arch Neurol, 63, 10, 1402-8, 2006.
11. Frutos-Alegría, M.T, Moltó Jordà JM, Morera-Guitart J, Sánchez-Pérez A, Ferrer-Navajas M. Perfil Neuropsicológico del deterioro cognitivo leve con afectación de múltiples áreas cognitivas. Importancia de la amnesia en la distinción de dos subtipos de pacientes. Rev Neurología 44, 8, 455-459, 2007.
12. Huang TL, Zandi PP, Tucker AL, et al., Benefits of fatty fish on dementia risk are stronger for those without APOE epsilon4. Neurology, 65, 9, 1409-14, 2005.
13. Kotani S, Sakaguchi E, Warashina S, Matsukawa N, Ishikura Y, Kiso Y, Sakakibara M. Dietary supplementation of arachidonic and docosahexaenoic acids improves cognitive dysfunction. Neurosci Res, 56, 2, 159-64, 2006.
14. Lobo A. Ezquerra J, Sala F. Seva J.M. Mini Examen Cognitivo; un test sencillo, práctico para detectar alteraciones intelectivas en pacientes médicos. Actas Luso Esp Neurol Psiquiatr Cienc Afines, 3, 189-202. 1979.
15. Lobo A. Saza P. Marcos G, Díaz J, De la Cámara C, Ventura T et al: Revalidación y normalización del Mini-Examen Cogniscitivo (primera versión en castellano del Mini-Mental Status Examination) en la población general geriátrica. Med Clin (Barc), 112, 767-74, 1999.
16. Morris MC, Evans DA, Bienias JL, Tangney CC, Bennett DA, Wilson RS, Aggarwal N. Consumption of fish and n-3 fatty acids and risk of incident Alzheimer Disease. Ach Neurol, 60, 940-946, 2003.
17. Okonkowo O.C, Griffith H.R, Copeland J.N:, Belue K., Lanza S. et al. Medical decision making capacity in mild cognitive impairment. Omega-3 year longitudinal study. Neurology, 71, Nov 4; 1474-1480, 2008.
18. Petersen R.C, Doody R, Kurz A, Mohs R.C. Morris JC et al. Current concepts in mild cognitive impairment. Archives of Neurology , 58, 1985-1992, 2001.
19. Salloway S. Buying Time: Management of Mild Cognitive Impairment and Early Dementia. International Psichogeriatrics, 18 (sup 1): 17-23, 2006 - resumen comité científico SIIC-.
20. Sarasola D., De Luján M., Sabe L., Caballero A., Manes F. Utilidad del *Addenbrooke’s Cognitive* *Examination* en Español para el Diagnóstico de Demencia y para la diferenciación entre Enfermedad de 18. Alheimer y la Demencia Frontotemporal. Rev. Arg. De Neuropsicol, 4, 1-11, 2004.
21. Schaefer EJ, Bongard V, Beiser AS, Lamon-Fava S, Robins SJ, Au R, Tucker KL, Kyle DJ, Wilson PW, Wolf PA. Plasma phosphatidylcholine docosahexaenoic acid content and risk of dementia and Alzheimer disease: the Framingham Heart Study. Arch Neurol, 63, 11, 1545-50, 2006.

Sheikh JI, Yesavage JA. Geriatric Depression Scale (GDS): Recent evidence and development of a shorter version. En: Brink TL eds. Clinical Gerontology: A guide to assessment and intervention. New York: Haworth Press, 165-173, 1986.

1. Solomon PR, Hirschoff A, Kelly B, Relin M, Brush M, de Veaux MD et al. A 7 minute neurocognitive screen battery highly sensitive to Alzheimer’s disease. Archives of Neurology, 55, 349-355, 1998.
2. Tully AM, Roche HM, Doyle R, Fallon C, Bruce I, Lawlor B, Coakley D, Gibney MJ. Low serum cholesteryl ester-docosahexaenoic acid levels in Alzheimer´s disease: a case-control study. British Journal of Nutrition, 89, 4, 483-489, 2003.
3. Vinyoles E., Vila J, Argimon J.M. et al. Concordancia entre el Mini-Examen Cognoscitivo y el Mini-Mental State Examination en el cribado del déficit cognitivo. Aten Primaria, 30, 1, 5-15, 2002.

Wechsler, D. WMS-III. Escala de Memoria de Wechsler III. Madrid: TEA Ediciones; 2004.

Yesavage JA, Brink TL, Rose TL. Development and validation of a geriatric depression screening scale: a preliminary report. J Psychiatry Res, 17, 37-49, 1983.

**3. UTILIDAD POTENCIAL DEL ESTUDIO**

Por lo anteriormente visto, diversos estudios epidemiológicos confirman que el consumo de ácidos grasos omega-3 se asocia a una reducción del riesgo de deterioro cognitivo. Parece que concentraciones más elevadas de acido docosahexaenoido (DHA) + ácido eicosapentaenoico (EPA) de las habituales en la población general produce un efecto de tipo protector frente al deterioro cognitivo.

Se han estudiado las posibles vías fisiológicas a través de las que podría tener efecto los ácidos omega-3: metabolitos neuroprotectores, reducción de metabolitos del ácido araquidónico y aumento de determinados factores neurotróficos. Algunas de estas vías también tendrían como efecto la reducción del riesgo de enfermedad cardiovascular.

Un conocimiento del efecto del complemento sobre las áreas cognitivas de los sujetos de edad avanzada con alteración de memoria nos permitiría introducir suplementos dietéticos favorecedores o aspectos preventivos específicos para estas personas con el objetivo de:

1. Realizar actividades preventivas con los pacientes con alteración de memoria por la edad dando un aporte suplementario de ácidos grasos omega-3.
2. Retrasar el inicio del deterioro cognitivo en estos sujetos.

***BIBLIOGRAFÍA***

- Morris MC, Evans DA, Tangney CH et al. Fish consumption and cognitive decline with age in a large community study. Arch Neurol, 62, 1849-1853, 2005.
- Cole GM et al. Omega 3 fatty acids and dementia. doi: 10.1016/j.plefa.2009.05.015
- Lim WS, Gammack JK et al. Ácidos grasos omega 3 para la prevención de la demencia (revisión Cochrane traducida). Biblioteca Cochrane Plus, 2007, 3 Chichester UK. John Wiley and sons Ltd
- Kotani S., Sakaguchi E., Warashina S., et al. Dietary supplementation of arachidonic and docosahexaenoic acids improves cognitive disfunction. Neuroscience research 56 (2006) 159-164.
- Petersen, R.C., 2004. Mild cognitive impairment as a diagnostic entity. Journal of Internal Medicine Sep;256, 183-194.
- Winblad B., Palmer K., Kivipelto M., Jelic V., . Fratiglioni. Mild cognitive impairment – beyond controversies, towards a consensus: report of the International Working Group on Mild Cognitive Impairment. Journal of Internal Medicine 2004; 256: 240–246
- Freund-Levi, M. Eriksdotter-Jo¨nhagen, T. Cederholm, H. Basun, G. Faxe´n-Irving, A. Garlind, Inger Vedin, MSci; Bengt Vessby, Lars-Olof Wahlund, J. Palmblad, W-3 Fatty Acid Treatment in 174 Patients With Mildto Moderate Alzheimer Disease: OmegAD Study.A Randomized Double-blind Trial ARCH Neurology 2006 vol 63, 1403-1408
- Whalley, H. C Fox, K. W Wahle, J. M Starr, I J Deary. Cognitive aging, childhood intelligence, and the use of food supplements: possible involvement of n_3 fatty acids1–3. Am J Clin Nutr 2004;80:1650 –1657.
- Kalmijn, M. P.J. van Boxtel, M. Ocké, W. M. M. Verschuren, D. Kromhout, L.J. Launer. Dietary intake of fatty acids and fish in relation to cognitive performance at middle age. Neurology 2004; 62:275-280
- Solfrizzi, C. Capurso, A. D’Introno, A.M. Colacicco, V. Frisardi,A. Santamato, M. Ranieri, P. Fiore, G. Vendemiale, D. Seripa, A. Pilotto,A. Capurso, F. Panza. Dietary fatty acids, age-related cognitive decline,and mild cognitive impairment The Journal of Nutrition, Health & Aging, 12, 6, 382-386, 2008.
- Schaefer, V. Bongard, A. S. Beiser, S. Lamon-Fava, S. J. Robins, R. Au, K. L. Tucker, D. J. Kyle, P. W. F. Wilson, P. A. Wolf. El contenido plasmático de ácido docosahexanoico en fosfatidilcolina y el riesgo de demencia y enfermedad de Alzheimer. Estudio cardiovascular de Framingham***.*** Arch neurology, 2006, 63, 1545-1550
- Fontani, F: Corradeschi, A: Felici, F. Alfatti, S. Migliorini, L.Lodi. Cognitive and physiological effects of Omega-3 polyunsaturated fatty acid supplementation in healthy subjects. European Journal of Clinical Investigation, 2005, 35, 691-699.
- Cole, Q. Ma, S.A. Frautschy. Ácidos grasos omega-3 y Demencia.Official Journal of the International Society for the Study of Fatty Acids and Lipid. (2009), doi:1 0.1016 /j. plefa.2009. 05.015.
- Yurko-Mauro, Deanna McCarthy, Dror Rom, Edward B. Nelson, Alan S. Ryan, Andrew Blackwell, Norman Salem, Mary Stedman. Beneficial effects of docosahexaenoic acid on cognition in age-related cognitive decline Alzheimer's & Dementia: The Journal of the Alzheimer's Association DOI: 10.1016/j.jalz.2010.01.013

#### 4. OBJETIVOS

**4.1. Objetivo principal**

Estudiar si la administración a lo largo de un año de un complemento en la dieta con ácidos grasos omega-3 y otras sustancias contenidas en el mismo, produce un mejor rendimiento cognitivo en los sujetos de edad avanzada sin déficit previo o con un déficit cognitivo ligero.

Para ello se utilizará la descripción de las variaciones en los cuestionarios: MEC de Lobo de 35 puntos, Fluencia Verbal, Test del Reloj, GDS de Reisberg y SPSMQ de Pfeiffer.

# **4.2. Objetivos secundarios**

- Estudiar las características de los sujetos que obtengan mejores resultados con el suplemento diferenciándolos de aquellos que no obtengan beneficio.
- Estudiar si hay cambios en otras áreas cognitivas: memoria, funciones ejecutivas, atención, lenguaje, velocidad de procesamiento.

**5. DISEÑO DEL ESTUDIO**

**5.1. Resumen**

Estudio multicéntrico, observacional, longitudinal, aleatorizado, frente a placebo.

Un grupo recibirá 3 cápsulas del complemento de ácidos grasos omega-3 del producto de referencia. Total: 1.050mg de DHA y 120 mg de EPA. Otro grupo recibirá placebo.

Se utilizará una muestra con selección por aleatorización mediante un programa informático en dos grupos “complemento alimenticio” y “placebo”. Los sujetos serán seleccionados en base a los criterios de inclusión y exclusión en las consultas de los centros gerontológicos participantes mediante el uso de la escala GDS de Reisberg (Estadíos 1, 2 y 3) y se incluirán en protocolo. Se eliminarán del estudio los sujetos que no sigan los tratamientos.

# **5.2. Metodología del estudio**

Sin alterar la práctica habitual ni el protocolo existente para estos pacientes en el Centro Gerontológico, se les aplicará una batería de cuestionarios de uso habitual.

Se incluirán pacientes sin deterioro cognitivo o con deterioro leve. Se pretende disponer al final del estudio de un mínimo de 150 (incluir 170) pacientes evaluables en total (75 suplemento alimenticio –incluir 85-; 75 placebo –incluir 85-).

Se realizará una primera entrevista por médicos y psicólogos. Todo el personal será entrenado en las pruebas que se administran para asegurar la fiabilidad inter-examinadores. La valoración incluye una exhaustiva historia clínica.

La adscripción al grupo control o experimental se hará, en el momento de incluir el paciente en el estudio, mediante randomización aleatoria con el uso de un programa informático y se disociarán los datos personales mediante hoja independiente (ANEXO 2)

A todos los sujetos candidatos se les informará del estudio y firmarán un consentimiento informado junto al profesional que realiza la adscripción (Visita 0).

El tiempo total de seguimiento será de 1 año (Visita 0) a contar desde el momento en que el paciente comience a tomar el suplemento o el placebo. Se realizará la valoración neuropsicológica y del estado de ánimo al inicio (Visita 0), a los 6 meses (Visita 1) y al año (Visita 2).

El complemento alimenticio será proporcionado por el laboratorio fabricante.

Un grupo tomará 3 cápsulas diarias del complemento de omega-3 comercializado en España: total mínimo de 75 pacientes –incluir 85-. Otro grupo similar será el grupo control al que se administrará placebo.

Al ser pacientes ingresados, tras la Visita 0 se les incluirá en su régimen alimenticio (al grupo con producto) una cápsula del complemento alimenticio en el desayuno, comida y cena. Total: 3 cápsulas; necesidad de 1080 cápsulas por paciente/año de estudio. Al grupo control se le administrará placebo.

**Se evaluará la evolución clínica de los pacientes mediante diferentes escalas:**

- **Escala de deterioro global. Global Deterioration Scale (GDS)**. Establece 7 estadíos posibles: 1 = normal, 2 = deterioro muy leve, 3= deterioro leve, 4= deterioro moderado, 5= deterioro moderadamente severo, 6= deterioro severo y 7 = deterioro muy severo. Describe cada estadío en términos operacionales y en base a un deterioro supuestamente homogéneo. Es una de las escalas más completas, simples y útiles para la estimación de la severidad de la demencia.

**Bibliografía**

- **Mini Mental State Examination** (MMSE) de Folstein y cols., versión validad al castellano por Lobo y col. Se basa en un cuestionario muy utilizado que explora las áreas: orientación temporal, orientación espacial, fijación, memoria, nominación, repetición, comprensión, lectura, escritura y dibujo.

Folstein, MF., Folstein, SE., McHugh, PR. Mini Mental State: A practice method for grading the cognitive state of patients for the clinician. Journal of Psychiatric Research, 12, 189-198, 1975.

Lobo A. Ezquerra J, Sala F. Seva J.M. Mini Examen Cognitivo; un test sencillo, práctico para detectar alteraciones intelectivas en pacientes médicos. Actas Luso Esp Neurol Psiquiatr Cienc Afines, 3, 189-202. 1979.

Lobo A. Saza P. Marcos G, Díaz J, De la Cámara C, Ventura T et al: Revalidación y normalización del Mini-Examen Cogniscitivo (primera versión en castellano del Mini-Mental Status Examination) en la población general geriátrica. Med Clin (Barc), 112, 767-74, 1999

- El **Cuestionario de actividad funcional** de **Pfeiffer (SPMSQ”),** que permite valorar el grado de autonomía para llevar a cabo tareas cotidianas tales como manejar dinero, hacer la compra, hacer la comida, entender y discutir noticias, salir a la calle solo, etc.

Pfeffer RI, Kurosaki TT, Harrah CH, Chance JM, Bates D, Detels R et al. A survey diagnostic tool for senile dementia. *Am J Epidemiol* 1981; 114: 515-7.

- **Fluencia verbal.** El test de fluencia verbal semántica, a pesar de su sencillez y facilidad de aplicación, se ha mostrado muy sensible al daño cerebral y en especial al deterioro cognitivo, formando parte de diversas baterías neuropsicológicas o utilizándose de forma aislada como ayuda al diagnóstico.

Rosen W.: "Verbal fluency in aging and dementia". J. Clin Neuropsychol 2: 135-146, 1980

- **Test del Reloj.** El Test del Dibujo del Reloj (TDR) es una prueba de detección sencilla, rápida y de fácil aplicación empleada tanto en la práctica clínica como en investigación para valorar el estado cognitivo del sujeto. Evalúa diferentes mecanismos implicados en la ejecución de la tarea, fundamentalmente funciones visoperceptivas, visomotoras y visoconstructivas, planificación y ejecución motoras. Según estos criterios, se ha establecido puntuaciones máximas.

Battersby, W.S., Bender, M.B., Pollack, M. y Kahn, R.L. (1956): Unilateral "spatial agnosia" ("inattention") in patients with cortical lesions. *Brain*, 79: 68-93.

Se adjunta esquema de procedimiento:

|  | **Día O**  **(Visita 0)** | **Mes 6**  **(Visita 1)** | **Mes 12**  **(Visita 2)** |
| --- | --- | --- | --- |
| **Consentimiento Informado** | **X** |  |  |
| **Escala GDS** | **X** |  | **X** |
| **MEC** | **X** | **X** | **X** |
| **Cuest. actividad funcional Pfeiffer** | **X** |  | **X** |
| **Frecuencia verbal** | **X** |  | **X** |
| **Test del reloj** | **X** |  | **X** |

Los datos de cada visita (Visita 0, Visita 1 y Visita 2) se recogerán en al correspondiente Hoja de Recogida de Datos (ANEXO 3)

**5.3. Variables**

- Demográficas (edad, sexo, estudios)
- Enfermedades orgánicas. Presencia o no de:

Alteraciones tiroideas

Enfermedad cardiovascular o cerebrovascular diagnosticada.

Factores de Riesgo Cardiovascular: HTA, DM, Obesidad, Tabaco, Dislipemia

- Depresión (se realizará anamnesis sobre su presencia)
- Consumo crónico de fármacos psicoactivos
- Participación en actividades de estimulación cognitiva

| Variable | Definición | Escala |
| --- | --- | --- |
| Edad | Edad del usuario en el momento del inicio del complemento | En años |
| Sexo |  | Hombre/ mujer |
| Estudios | Nivel de estudios | Analfabeto  Analfabeto funcional  Primarios  Bachiller  Medios  Universitarios |
|  | Años de estudios | En años |
| Profesión anterior habitual |  | Dirección de empresas  Técnico/ profesional  Comercio  Trabajador cualificado  Trabajador no cualificado  Ama de casa |
| Enfermedades | Presencia o no de:  Alteraciones tiroideas  Enfermedad cardiovascular o cerebrovascular diagnosticada.  Factores de Riesgo Cardiovascular: HTA, DM, Obesidad, Tabaco, Dislipemia | SI /NO |
| Trastorno del ánimo | Depresión | SI /NO |
| Consumo de fármacos | Fármacos psicoactivos tomados de forma crónica según tipos: Benzodiacepinas o ansiolíticos, hipnóticos, antidepresivos, neurolépticos, otros psicofármacos | SI /NO |
| GDS | Estadiaje deterioro cognitivo | 1-7 |
| MEC | Rendimiento cognitivo general | 0-35 |
| Cuestionario actividad funcional Pfeiffer | Memoria actual | Más de 5 = problema |
| Fluencia verbal | Funciones ejecutivas | < 13 en 1 min = problema |
| Test Reloj | Praxias, visoconstrucción , FFEE | 0-10 |

**5.2.1 Determinaciones previas**

Siguiendo el protocolo habitual en las Unidades de Demencia, los pacientes dispondrán en su historia clínica de información previa a su inclusión. Estos datos serán tenidos en cuenta para valorar los Criterios de Exclusión

**5.2.2. Suministro del complemento alimenticio**

Debido a que son pacientes ingresados, en el grupo con producto, se suministrará éste en cada una de las comidas principales (3 al día) precisándose por paciente un total de 1080 cápsulas durante el año de estudio a razón de 90 cápsulas/paciente/mes. Al grupo control se le administrará placebo.

# **5.3. Variables a valorar**

Para la evaluación de los pacientes se utilizarán; Escala Global del Deterioro (GDS) de Reisberg, MEC de Lobo, Cuestionario de Pfeiffer, Frecuencia Verbal y Test del Reloj.

**5.4. Definición de la población de estudio: criterios de selección**

**5.4.1. Criterios de inclusión**

- Pacientes de edad igual o mayor de 80 años.
- Pacientes sin deterioro cognitivo o con deterioro leve, seleccionados mediante escalas cognitivas teniendo en cuenta el informe de la persona, de sus familiares y el juicio global del examinador. Criterio: Global Deterioration Scale GDS (Escala de deterioro global) de Reisberg estadío menor de 4 (GDS 1,2 y 3)
- Que acepten entrar en el estudio y firmen el consentimiento informado.
- Los sujetos con enfermedades sistémicas se incluirán si se juzga que están controladas y que esta enfermedad no produce durante el estudio alteración de tipo cognitivo en el sujeto. Las alteraciones sensoriales (de la visión y audición) se incluirán si se juzga que no son graves y por lo tanto no comprometen los resultados.

**5.4.2. Criterios de exclusión**

- Nivel cultural que impida la comprensión, realización o valoración de los test aplicados.
- Tener enfermedades neurológicas, u otras alteraciones sistémicas o trastornos mentales mal controlados. Este dato es obtenido por historia clínica.
- Valoración negativa por parte del investigador a la vista de las pruebas habituales previas.
- Diagnóstico de Demencia establecida
- Sujetos que a lo largo del estudio sufran una disminución brusca del rendimiento funcional de 2 estadíos en la escala GDS de Reisberg, de forma permanente, atribuible a una enfermedad o acontecimiento vital agudo.
- Sujetos con antecedentes de epilepsia, crisis comiciales o convulsuiones

#### 5.5. Periodo de observación

La duración del periodo de recogida de datos será de 12 meses, desde la visita basal o Visita 0 a la final o Visita 2, con evaluación a los 6 meses (Visita 1).

#### 5.6. Predeterminación del tamaño muestral

Existen numerosas discrepancias con respecto a la evolución natural del deterioro cognitivo al año. Todo ello depende del cuestionario utilizado y de la edad.

Si aceptamos que las personas mayores de 80 años con deterioro cognitivo presentan en un 20% en evolución natural a la progresión y que con la intervención ésta puede ser solo del 5%, con una mejora intergrupos del 15% y asumimos un riesgo α= 0,05, y un riesgo un riesgo β= 0,20, el tamaño muestral sería de 75 pacientes por grupo. Total: 150 a evaluar. Presumiendo pérdidas de alrededor del 10%, deberíamos incluir con intención a tratar 170 pacientes, dos grupos de 85. El cálculo se ha efectuado con la aplicación informática EPICAL 2000 para diferencias de proporciones.

**6. PLAN DE ANÁLISIS ESTADÍSTICO**

Para el estudio de las diferencias entre los grupos control y experimental se realizará ANOVA, modelo lineal general con medidas repetidas, o diferencia de medias. Se controlará: edad, sexo, depresión y otras variables descriptivas que se consideren oportunas. El factor intra-sujetos será el tiempo (1ª evaluación, corte a los seis meses y última) y el factor inter-sujetos será el grupo al que se ha adscrito (control o experimental).

Se calculará el riego relativo. El tamaño del efecto se estudiará con el estadístico ETA.

Para estudiar los predictores de mejora realizamos regresión logística; el tamaño del efecto se calcula con el estadístico ODDS Ratio. Se utilizará un paquete estadístico SPSS 19 IBM.

**7. ASPECTOS ÉTICOS**

El investigador deberá realizar el estudio de acuerdo con los principios de la Declaración de Helsinki referente a la investigación médica en seres humanos. Las copias pueden ser obtenidas a través de la página web de la Asociación Médica Mundial (*World Medical Association*) en <http://www.wma.net/e/policy/b3.htm>.

El estudio deberá desarrollarse de acuerdo con el protocolo asegurando el cumplimiento de las normas de Buena Práctica Clínica.

**7.1. Evaluación beneficio-riesgo para los sujetos de la investigación**

El desarrollo del estudio no comporta riesgos añadidos al paciente, excepto los propios del manejo de su patología en la práctica clínica habitual.

**7.2. Hoja de información y formulario de consentimiento**

En cumplimiento de lo establecido en la declaración de Helsinki, será responsabilidad del investigador/colaborador informar al paciente sobre su participación en este proyecto. La participación es voluntaria y no supone ningún cambio ni en su tratamiento ni en su atención médica respecto a los que recibiría de no participar. El paciente recibirá información adecuada y se obtendrá su consentimiento por escrito antes de revisar su historia clínica y ser incluido en el estudio (ANEXO 4 y ANEXO 5).

**7.3. Confidencialidad de los datos**

Los datos de carácter personal del estudio serán datos disociados. Se seguirá asimismo lo establecido en la Ley Orgánica 15/1999 de 13 de diciembre, de “Protección de Datos de Carácter Personal”, para el manejo de datos personales disociados.

En el formulario de recogida de datos el paciente será identificado sólo mediante un código. El médico investigador mantendrá un registro confidencial que relacione los códigos de identificación con la identificación del paciente (ANEXO 2)

**7.4. Interferencia con los hábitos de prescripción del médico**

El presente estudio se desarrolla dentro de la práctica clínica habitual incluyéndose únicamente un complemento alimenticio a base de ácidos grasos poliinsaturados Omega-3 en el grupo experimental y placebo en el grupo control.

**8. ASPECTOS ORGANIZATIVOS**

1. Elaboración y presentación del proyecto para tesis
2. Presentación del proyecto y protocolo al Comité de Ética
3. Recogida progresiva de sujetos que cumplan los criterios de inclusión y de exclusión
4. Información a los pacientes y firma del consentimiento informado
5. Distribución aleatoria de los sujetos: control / experimental. Aleatorización informárica.
6. Comienzo de la complementación alimenticia y placebo
7. Valoraciones neuropsicológicas y conductuales

(V0, V1 y V2)Introducción en el ordenador de datos

1. Análisis de los datos. Comprobación de la hipótesis.
2. Elaboración de resultados finales
3. Elaboración de la tesis y posibilidad de publicación

**9. INFORME FINAL**

El informe final de resultados se realizará en un máximo de seis meses del final del estudio observacional.

**9.1. Difusión de los resultados**

Los resultados se utilizarán para la obtención del grado de Doctor y se hará su difusión por medio de publicaciones científicas, congresos especializados u otros medios específicos. La difusión de los informes clínicos del estudio será independiente de los resultados obtenidos.

**10. ANEXOS**

ANEXO 1: Descripción del complemento alimenticio a base de omega-3

ANESO 2: Hoja de Disociación de Datos

ANEXO 3: Hoja de Recogida de Datos

ANEXO 4: Test y escalas a plicar

ANEXO 4: Hoja e Consentimiento Informado

ANEXO 5: Hoja de Información al Paciente

ANEXO 6: Conformidad del CEIC

**ANEXO 1. Suplemento omega-3 (ACUTIL)**

## Complemento alimenticio de ácidos grasos poliinsaturados Omega-3, Ginkgo biloba, fosfatidilserina, vitamina E, vitamina B_12_ y ácido fólico.

El aceite de pescado, fuente natural de ácidos grasos poliinsaturados de la serie Omega 3 (EPA y DHA), asociado a una dieta equilibrada, puede influir favorablemente en la reducción de lipoproteínas de baja densidad (LDL) lo cual puede mejorar tanto el funcionamiento cardiovascular como el cognitivo.

Las hojas del árbol Ginkgo biloba contienen sustancias cuyos principales efectos parecen estar relacionados con sus propiedades antioxidantes, contribuyendo al funcionamiento de los mecanismos celulares frente a la oxidación.

La fosfatidilserina, pertenece a una clase de compuestos químicos conocidos como fosfolípidos*.* La fosfatidilserina interviene en el mantenimiento de la integridad estructural de la membrana celular.

La vitamina E, que se concentra a nivel de las membranas celulares, ayuda a regularizar numerosas reacciones metabólicas.

La vitamina B_12_ es una vitamina hidrosoluble, que favorece la formación de las células sanguíneas y de las neuronas.

El ácido fólico, una vitamina del complejo B, participa en procesos de metilación que son esenciales para el desarrollo neuronal.

**Ingredientes**:

Aceite de pescado con ácidos grasos poliinsaturados Omega-3; cápsula de gelatina (agente de recubrimiento: gelatina; humectantes: glicerol, sorbitol; colorante: E-172); extracto estandarizado de hojas de Ginkgo biloba (6%); emulgente: monoestearato de glicerilo; fosfatidilserina*; D-alfa tocoferol; cianocobalamina; ácido teroilmonoglutámico.

*Procedente de la soja.

| INFORMACIÓN NUTRICIONAL | Por dosis diaria  (1 cápsula) | % CDR por 1 cps |
| --- | --- | --- |
| Ácidos grasos poliinsaturados Omega-3 | 350 mg | - |
| de los cuales EPA* - ácido eicosapentaenoico | 40 mg | - |
| DHA* - ácido docosahexaenoico | 250 mg | - |
| Fosfatidilserina | 15 mg | - |
| Vitamina E | 5 mg α-TE | 50 |
| Vitamina B_12_ | 5 μg | 500 |
| Ácido fólico | 250 μg | 125 |

CDR: Cantidad Diaria Recomendada

*: Valores medios

| APORTE DE COMPONENTES VEGETALES | Por dosis diaria  (1 cápsula) |
| --- | --- |
| Extracto estandarizado de hojas de Ginkgo biloba | 60 mg |
| proporcionando  24 % de glucósidos ginkgoflavónicos | 14,5 mg |
| 6% de ginkgólidos y bilobálidos | 3,6 mg |

Modo de empleo:

Tomar 1 cápsula al día junto con la comida o bebida. Si toma este producto por primera vez, puede doblar la dosis durante las primeras 12 semanas.

**Advertencias**:

Conservar en lugar fresco y seco. Mantener fuera del alcance de los niños. Los complementos alimenticios no deben utilizarse como sustitutos de una dieta equilibrada. No superar la dosis diaria recomendada.

#### Presentación: 30 cápsulas

**Fabricado por**:

Efamol Ltd

14 The Mole Business Park

Leatherhead, Surrey

KT22 7BA – REINO UNIDO

**Comercializado por**:

ANGELINI FARMACÉUTICA, S.A.

C. Osi, 7 - 08034 Barcelona

**ANEXO 2. Hoja de disociación de datos**

| | Nº de disociación | Iniciales/  H Clínica |  | Nº de disociación | Iniciales/  H Clínica | | --- | --- | --- | --- | --- | | 1 |  |  | 31 |  | | 2 |  |  | 32 |  | | 3 |  |  | 33 |  | | 4 |  |  | 34 |  | | 5 |  |  | 35 |  | | 6 |  |  | 36 |  | | 7 |  |  | 37 |  | | 8 |  |  | 38 |  | | 9 |  |  | 39 |  | | 10 |  |  | 40 |  | | 11 |  |  | 41 |  | | 12 |  |  | 42 |  | | 13 |  |  | 43 |  | | 14 |  |  | 44 |  | | 15 |  |  | 45 |  | | 16 |  |  | 46 |  | | 17 |  |  | 47 |  | | 18 |  |  | 48 |  | | 19 |  |  | 49 |  | | 20 |  |  | 50 |  | | 21 |  |  | 51 |  | | 22 |  |  | 52 |  | | 23 |  |  | 53 |  | | 24 |  |  | 54 |  | | 25 |  |  | 55 |  | | 26 |  |  | 56 |  | | 27 |  |  | 57 |  | | 28 |  |  | 58 |  | | 29 |  |  | 59 |  | | 30 |  |  | 60 |  | |
| --- | --- | --- | --- | --- | --- | --- | --- | --- | --- | --- | --- | --- | --- | --- | --- | --- | --- | --- | --- | --- | --- | --- | --- | --- | --- | --- | --- | --- | --- | --- | --- | --- | --- | --- | --- | --- | --- | --- | --- | --- | --- | --- | --- | --- | --- | --- | --- | --- | --- | --- | --- | --- | --- | --- | --- | --- | --- | --- | --- | --- | --- | --- | --- | --- | --- | --- | --- | --- | --- | --- | --- | --- | --- | --- | --- | --- | --- | --- | --- | --- | --- | --- | --- | --- | --- | --- | --- | --- | --- | --- | --- | --- | --- | --- | --- | --- | --- | --- | --- | --- | --- | --- | --- | --- | --- | --- | --- | --- | --- | --- | --- | --- | --- | --- | --- | --- | --- | --- | --- | --- | --- | --- | --- | --- | --- | --- | --- | --- | --- | --- | --- | --- | --- | --- | --- | --- | --- | --- | --- | --- | --- | --- | --- | --- | --- | --- | --- | --- | --- | --- | --- | --- | --- | --- | --- |

| Nº de disociación | Iniciales/  H Clínica |  | Nº de disociación | Iniciales/  H Clínica |
| --- | --- | --- | --- | --- |
| 61 |  |  | 91 |  |
| 62 |  |  | 92 |  |
| 63 |  |  | 93 |  |
| 64 |  |  | 94 |  |
| 65 |  |  | 95 |  |
| 66 |  |  | 96 |  |
| 67 |  |  | 97 |  |
| 68 |  |  | 98 |  |
| 69 |  |  | 99 |  |
| 70 |  |  | 100 |  |
| 71 |  |  | 101 |  |
| 72 |  |  | 102 |  |
| 73 |  |  | 103 |  |
| 74 |  |  | 104 |  |
| 75 |  |  | 105 |  |
| 76 |  |  | 106 |  |
| 77 |  |  | 107 |  |
| 78 |  |  | 108 |  |
| 79 |  |  | 109 |  |
| 80 |  |  | 110 |  |
| 81 |  |  | 111 |  |
| 82 |  |  | 112 |  |
| 83 |  |  | 113 |  |
| 84 |  |  | 114 |  |
| 85 |  |  | 115 |  |
| 86 |  |  | 116 |  |
| 87 |  |  | 117 |  |
| 88 |  |  | 118 |  |
| 89 |  |  | 119 |  |
| 90 |  |  | 120 |  |

| Nº de disociación | Iniciales/  H Clínica |  | Nº de disociación | Iniciales/  H Clínica |
| --- | --- | --- | --- | --- |
| 122 |  |  | 152 |  |
| 123 |  |  | 153 |  |
| 124 |  |  | 154 |  |
| 125 |  |  | 155 |  |
| 126 |  |  | 156 |  |
| 127 |  |  | 157 |  |
| 128 |  |  | 158 |  |
| 129 |  |  | 159 |  |
| 130 |  |  | 160 |  |
| 131 |  |  | 161 |  |
| 132 |  |  | 162 |  |
| 1331 |  |  | 163 |  |
| 134 |  |  | 164 |  |
| 135 |  |  | 165 |  |
| 136 |  |  | 166 |  |
| 137 |  |  | 167 |  |
| 138 |  |  | 168 |  |
| 139 |  |  | 169 |  |
| 140 |  |  | 170 |  |
| 141 |  |  |  |  |
| 142 |  |  |  |  |
| 143 |  |  |  |  |
| 144 |  |  |  |  |
| 145 |  |  |  |  |
| 146 |  |  |  |  |
| 147 |  |  |  |  |
| 148 |  |  |  |  |
| 149 |  |  |  |  |
| 150 |  |  |  |  |
| 151 |  |  |  |  |

**ANEXO 3. Hoja de Recogida de datos**

Fecha valoración: ___/___/______

Código de centro Nº de disociación Nº interno

**DATOS SOCIODEMOGRÁFICOS E HISTORIA CLÍNICA**

| F. Nacimiento | __ / __ / __ | | | | Edad: | | ___ años | | | Sexo: | | 1 ⬜ V | | | |  | Años escolarización: _____ | | |
| --- | --- | --- | --- | --- | --- | --- | --- | --- | --- | --- | --- | --- | --- | --- | --- | --- | --- | --- | --- |
|  | | |  | | |  | | |  | | | 2 ⬜ M | | |  | |  | |  |
| Nivel de estudios | | 1 ⬜ Analfabeto | | | | | | | 3 ⬜ Estudios primarios | | | | | | | | 5 ⬜ Estudios medios | | |
|  | | 2 ⬜ Analfabeto funcional | | | | | | | 4 ⬜ Bachiller elemental | | | | | | | | 6 ⬜ Estudios superiores | | |
|  | | | |  | | | | | | | | | |  | | | | | |
| Profesión | | 1 ⬜ Dirección de empresas | | | | | | | 3 ⬜ Comercio | | | | | | | | 5 ⬜ Trabajador no cualificado | | |
|  | | 2 ⬜ Técnico / profesional | | | | | | | 4 ⬜ Trabajador cualificado | | | | | | | | 6 ⬜ Ama de casa | | |
|  | | | |  | | | | | | | | | |  | | | | | |
| Ant. personales | | 1 ⬜ Enf. cardiovascular | | | | | | | 6 ⬜ Alteraciones tiroideas | | | | | | | | 10 ⬜ Enf. psiquiátricas | | |
|  | | 2 ⬜ Hipertensión | | | | | | | 7 ⬜ Parkinson | | | | | | | | ⬜ Depresión | | |
|  | | 3 ⬜ Diabetes | | | | | | | 8 ⬜ Tabaquismo | | | | | | | | ⬜ Ansiedad | | |
|  | | 4 ⬜ Dislipemias | | | | | | | 9 ⬜ Alcoholismo | | | | | | | | ⬜ Otras | | |
|  | | 5 ⬜ Ictus | | | | | | |  | | | | | | | |  | | |
|  | | | |  | | | | | | | | | |  | | | | | |
| Tto. farmacológico | | 1 ⬜ BZD/ansiolíticos/hipnóticos | | | | | | | 5 ⬜ Antiepilépticos | | | | | | | |  | | |
|  | | 2 ⬜ Antidepresivos | | | | | | | 6 ⬜ Otros psicofármacos | | | | | | | |  | | |
|  | | 3 ⬜ Neurolépticos | | | | | | |  | | | | | | | |  | | |
|  | | 4 ⬜ Antiparkinsonianos | | | | | | |  | | | | | | | |  | | |
|  | | | |  | | | | | | | | | |  | | | | | |
|  | |  | | | | | |  | | | | |  | | | | |  | |
|  | |  | | | | | | | | | | | |  | | | | | |
| ¿Realiza grupo de estimulación cognitiva? | | | | | | | | 1 ⬜ SÍ | | | 2 ⬜ NO | | | | | | | | |

**RESULTADOS DE LAS PRUEBAS**

| **Test** | **Visita 0** | **Visita 1** | **Visita3** |
| --- | --- | --- | --- |
| **MEC** |  |  |  |
| **Cuestionario Pfeffer** |  |  |  |
| **Frecuencia verbal** |  |  |  |
| **Test del reloj** |  |  |  |
| **GDS Reisberg** |  |  |  |

Efectos adversos (detallar tipo, fecha supresión o no del producto)

**ORIENTACIÓN**

**ANEXO 4. Test y escalas a aplicar**

Fecha valoración: ___/___/______

**MINI EXAMEN**

**COGNOSCITIVO (MEC)**

Paciente.......................................................................................Edad................

Ocupación......................................................Escolaridad....................................

Examinado por..................................................Fecha..........................................

**ORIENTACIÓN**

• Dígame el día...........fecha ……..Mes.......................Estación...........................Año..........

­**___5**

• Dígame el hospital (o lugar).............................................................................

planta.....................ciudad.................Provincia......................Nación................

**___5**

**FIJACIÓN**

• Repita estas tres palabras ; peseta, caballo, manzana (hasta que se las aprenda)

**­­___3**

**CONCENTRACIÓN Y CÁLCULO**

• Si tiene 30 ptas. y me dando de tres en tres ¿cuantas le van quedando ?

**___5**

• Repita estos tres números : 5,9,2 (hasta que los aprenda) .Ahora hacia atrás

**___3**

**MEMORIA**

• ¿Recuerda las tres palabras de antes ?

**___3**

**LENGUAJE Y CONSTRUCCIÓN**

• Mostrar un bolígrafo. ¿Qué es esto ?, repetirlo con un reloj

**___2**

• Repita esta frase : En un trigal había cinco perros

**___1**

• Una manzana y una pera ,son frutas ¿verdad ?

¿qué son el rojo y el verde ?

**­­___2**

• ¿Que son un perro y un gato ?

**___3**

• Coja este papel con la mano derecha dóblelo y póngalo encima de la mesa

**­­___1**

• Lea esto y haga lo que dice : CIERRE LOS OJOS

**___1**

• Escriba una frase

**­­___1**

• Copie este dibujo**___1**

***Puntuación máxima 35.***

***Punto de corte* Adulto no geriátricos 24**

**Adulto geriátrico 20**

**APLICACIÓN DEL TEST**

**Orientación:**

Seguir las indicaciones del test (un punto cada respuesta correcta).

**Fijación:**

Repetir claramente cada palabra en un segundo. Se le dan tantos puntos como palabras repite correctamente al primer intento. Hacer hincapié en que lo recuerde ya que más tarde se le preguntará.

**Concentración:**

Si no entiende o se resiste, se le puede animar un poco, como máximo reformular la pregunta como sigue: “ *si tiene 30 euros y me da 3 ¿Cuántos euros le quedan?*  *y a continuación siga dándome de 3 en 3* ( sin repetir la cifra que él dé). Un punto por cada substracción correcta, exclusivamente. Por ejemplo 30 menos 3 = 28 (0 puntos); si la siguiente substracción es de 25, ésta es correcta (1 punto). Repetir los dígitos lentamente: 1 segundo cada uno hasta que los aprenda. Después pedirle que los repita en orden inverso y se le da un punto por cada dígito que coloque en oposición inversa correcta.

Por ejemplo, 592 (lo correcto es 295); si dice 925 le corresponde 1 punto.

**Memoria:**

Seguir las instrucciones del test, dando amplio margen de tiempo para que pueda recordar, pero sin ayudarle ( un punto por cada palabra recordada)

**Lenguaje y construcción:**

Seguir las instrucciones puntualizando que:

- Leerle la frase despacio y correctamente articulada. Para concederle 1 punto tiene que ser repetida a la primera y correctamente articulada, un fallo en una letra es 0 puntos.
- Semejanzas; para darle un punto en verde-rojo tiene que responder inexcusablemente “colores”. Para la semejanza perro-gato la contestación correcta exclusiva es animales o animales de “x” características o bichos.
- En la ordenes verbales, si coge el papel con la mano izquierda es un fallo en ese apartado. Si lo dobla más de dos veces otro fallo. Dependiendo de la posición del paciente se podrá modificar la orden de poner el papel en la mesa o en el suelo. Cada una de las partes de la orden ejecutada correctamente es un punto, hasta un máximo de 3.
- Para los test de la lectura y escritura, pedir al paciente que se coloque sus gafas, si las usa, y si es preciso escribir la orden y los pentágonos en trazos grandes en la parte posterior del papel , para que los vea perfectamente. Se le concede un punto si, independientemente de lo lea en voz alta, cierra los ojos sin que se le insista verbalmente. Recalcar antes, dos veces como máximo que lea y haga lo que pone en el papel.
- Para escribir una frase instruirle que no sea su nombre. Si es necesario puede usarse un ejemplo, pero insistiendo que tiene que escribir algo distinto. Se requiere sujeto, verbo y complemento para dar un punto (las frases impersonales sin sujeto)
- Figuras: la ejecución correcta (1 punto) requiere que cada pentágono tenga exactamente 5 lados y 5 ángulos y tienen que estar entrelazados entre sí con dos puntos de contacto.

*Mini-Examen Cognoscitivo. Versión española del Mini-Mental Status Examination de Folstein et al (1975). Validado por Lobo et al (1979-94)*

*Publicación más representativa :* un sencillo, práctico, para detectar alteraciones intelectuales en paciente médicos. Actas Luso-Españolas de Neurología, psiquiatría y ciencias afines vol 3, 189-202, 1979

**Short Portable Mental State Questionnaire (SPMSQ) de Pfeiffer**

Muy rápido y sencillo de utilizar para "screening" por parte del médico de Atención Primaria

| **SPMSQ de Pfeiffer** | |
| --- | --- |
| **Pregunta a realizar** | **Errores** |
| Total (máximo: 10 puntos) |  |
| ¿Qué fecha es hoy? (día, mes y año) |  |
| ¿Qué día de la semana es hoy? |  |
| ¿Dónde estamos ahora? (lugar o edificio) |  |
| ¿Cuál es su número de teléfono? (o su dirección si no tiene teléfono) |  |
| ¿Qué edad tiene? |  |
| ¿Cuándo nació? (día, mes y año) |  |
| ¿Cómo se llama el Presidente del Gobierno? |  |
| ¿Cómo se llamaba el anterior Presidente del Gobierno? |  |
| ¿Cuál es el primer apellido de su madre? |  |
| Reste de tres en tres desde veinte |  |

Se adjudica un punto por cada error, considerando erróneo un ítem por un solo fallo en cualquiera de sus partes si las tiene. Por ejemplo, un solo fallo en la secuencia de restas hará errónea la serie y dará por lo tanto un punto más al total. Lo mismo pasa con las fechas: simplemente conque no acierte el mes, el día o el año, se considerará como errónea la fecha, aunque acierte sus otros dos componentes.

Se considera patológico un total de 5 o más puntos, permitiéndose un error de más en caso de no haber recibido el paciente estudios primarios, o un error de menos si ha recibido estudios superiores.

**TEST FLUENCIA VERBAL**

**Dígame todos los animales que conozca.**

Se cronometra todos los que dice en un minuto. Si dice menos de 13 sospechamos deterioro cognitivo

El punto de corte puede varíar según las características de la población y la sensibilidad y especificidad que se quiera conseguir

La disminución de la espontaneidad verbal y la dificultad en la fluidez pueden evaluarse mediante este instrumento. Esta prueba mide el número de elementos de una categoría que puede evocar un sujeto en un tiempo determinado, habitualmente un minuto; se trata de una tarea compleja desde el punto de vista cognitivo, en la que intervienen procesos lingüísticos, mnésicos y ejecutivos.

Es un test de muy fácil y rápida aplicación, que no precisa material alguno y que puede ser aplicado a sujetos que son intestables por otros medios, analfabetos, déficits sensoriales como ceguera, déficits motores, etc), o en situaciones en las que otras pruebas son difíciles o imposibles de realizar como en el caso del enfermo encamado. El TVFs es muy sensible a todo tipo de daño cerebral, afectándose precozmente en procesos que condicionan deterioro cognitivo y en especial en la Enfermedad de Alzheimer. El entorno de aplicación de la prueba dictará siempre el punto de corte óptimo, en función de si queremos minimizar los falsos negativos, estudios poblacionales, screening) o los falsos positivos, uso del test como prueba de confirmación:

  El TFVs sería ideal para aplicarlo en estudios de screening y poblacionales eliminando de una segunda fase a aquellos sujetos que puntúen por encima del punto de corte; mejorándose el resultado si se elige un punto de corte que mejore la sensibilidad (<13) a expensas de un mayor número de falsos positivos.

  Así mismo, puede ser un excelente test para confirmar la presencia de demencia en sujetos en los que ésta se sospecha sobre la base de un test previo como el MMSE; en este sentido, se puede elegir un punto de corte con mayor especificidad (<9), con lo que los falsos negativos serían prácticamente inexistentes.

**GDS de Reisberg**

Global Deterioration Scale (Escala de deterioro global) de Reisberg

**GDS-1, ausencia de alteración cognitiva**

([Miniexamen cognoscitivo -MEC- de Lobo](http://www.hipocampo.org/lobo.asp) entre 30 y 35 puntos). Se corresponde con el individuo normal:

Ausencia de quejas subjetivas.

Ausencia de trastornos evidentes de la memoria en la entrevista clínica.

**GDS-2, disminución cognitiva muy leve**

([MEC de Lobo](http://www.hipocampo.org/lobo.asp) entre 25 y 30 puntos). Se corresponde con el deterioro cognitivo subjetivo:

Quejas subjetivas de defectos de memoria, sobre todo en:

a) Olvido de dónde ha colocado objetos familiares.

b) Olvido de nombres previamente bien conocidos.

No hay evidencia objetiva de defectos de memoria en el examen clínico.

No hay defectos objetivos en el trabajo o en situaciones sociales.

Hay pleno conocimiento y valoración de la sintomatología.

**GDS-3, defecto cognitivo leve**

([MEC de Lobo](http://www.hipocampo.org/lobo.asp) entre 20 y 27 puntos). Se corresponde con el deterioro cognitivo leve:

Primeros defectos claros: manifestaciones en una o más de estas áreas:

a) El paciente puede haberse perdido en un lugar no familiar.

b) Los compañeros detectan rendimiento laboral pobre.

c) Las personas más cercanas detectan defectos en la evocación de palabras y nombres.

d) Al leer un párrafo de un libro retiene muy poco material.

e) Puede mostrar una capacidad muy disminuida en el recuerdo de las personas nuevas que ha conocido.

f) Puede haber perdido o colocado en un lugar erróneo un objeto de valor.

g) En la exploración clínica puede hacerse evidente un defecto de concentración.

Un defecto objetivo de memoria únicamente se observa con una entrevista intensiva.

Aparece un decremento de los rendimientos en situaciones laborales o sociales exigentes.

La negación o desconocimiento de los defectos se hace manifiesta en el paciente.

Los síntomas se acompañan de ansiedad discreta-moderada.

**GDS-4, defecto cognitivo moderado**

([MEC de Lobo](http://www.hipocampo.org/lobo.asp) entre 16 y 23 puntos). Se corresponde con una demencia en estadio leve:

Defectos claramente definidos en una entrevista clínica cuidadosa en las áreas siguientes:

a) Conocimiento disminuido de los acontecimientos actuales y recientes.

b) El paciente puede presentar cierto déficit en el recuerdo de su propia historia personal.

c) Defecto de concentración puesto de manifiesto en la sustracción seriada de sietes.

d) Capacidad disminuida para viajes, finanzas, etc.

Frecuentemente no hay defectos en las áreas siguientes:

a) Orientación en tiempo y persona.

b) Reconocimiento de personas y caras familiares.

c) Capacidad de desplazarse a lugares familiares.

Incapacidad para realizar tareas complejas.

La negación es el mecanismo de defensa dominante.

Disminución del afecto y abandono en las situaciones más exigentes.

**GDS-5, defecto cognitivo moderado-grave**

([MEC de Lobo](http://www.hipocampo.org/lobo.asp) entre 10 y 19 puntos). Se corresponde con una demencia en estadio moderado:

El paciente no puede sobrevivir mucho tiempo sin alguna asistencia.

No recuerda datos relevantes de su vida actual: su dirección o teléfono de muchos años, los nombres de familiares próximos (como los nietos), el nombre de la escuela, etc.

Es frecuente cierta desorientación en tiempo (fecha, día de la semana, estación, etc.) o en lugar.

Una persona con educación formal puede tener dificultad contando hacia atrás desde 40 de cuatro en cuatro, o desde 20 de dos en dos.

Mantiene el conocimiento de muchos de los hechos de mayor interés concernientes a sí mismo y a otros.

Invariablemente sabe su nombre, y generalmente el de su esposa e hijos.

No requiere asistencia en el aseo ni en la comida, pero puede tener cierta dificultad en la elección de los vestidos adecuados

**GDS-6, defecto cognitivo grave**

([MEC de Lobo](http://www.hipocampo.org/lobo.asp) entre 0 y 12 puntos). Se corresponde con una demencia en estadio moderadamente grave:

Ocasionalmente puede olvidar el nombre de la esposa, de la que, por otra parte, depende totalmente para sobrevivir.

Desconoce los acontecimientos y experiencias recientes de su vida.

Mantiene cierto conocimiento de su vida pasada, pero muy fragmentario.

Generalmente desconoce su entorno, el año, la estación, etc.

Puede ser incapaz de contar desde 10 hacia atrás, y a veces hacia adelante.

Requiere cierta asistencia en las actividades cotidianas.

Puede tener incontinencia o requerir ayuda para desplazarse, pero puede ir a lugares familiares.

El ritmo diurno está frecuentemente alterado.

Casi siempre recuerda su nombre.

Frecuentemente sigue siendo capaz de distinguir entre las personas familiares y no familiares de su entorno.

Cambios emocionales y de personalidad bastante variables, como:

a) Conducta delirante: puede acusar de impostora a su esposa, o hablar con personas inexistentes, o con su imagen en el espejo.

b) Síntomas obsesivos, como actividades repetitivas de limpieza.

c) Síntomas de ansiedad, agitación e incluso conducta violenta, previamente inexistente.

d) Abulia cognitiva, pérdida de deseos, falta de elaboración de un pensamiento para determinar un curso de acción propositivo.

**GDS-7, defecto cognitivo muy grave**

([MEC de Lobo](http://www.hipocampo.org/lobo.asp) = 0 puntos, impracticable). Se corresponde con una demencia en estadio grave:

Pérdida progresiva de las capacidades verbales. Inicialmente se pueden verbalizar palabras y frases muy circunscritas; en las últimas fases no hay lenguaje, únicamente gruñidos.

Incontinencia de orina. Requiere asistencia en el aseo y en la alimentación.

Se van perdiendo las habilidades psicomotoras básicas, como la deambulación.

El cerebro es incapaz de decir al cuerpo lo que ha de hacer. Frecuentemente aparecen signos y síntomas neurológicos generalizados y corticales.

Nota aclaratoria: La correlación entre el estadio GDS y la puntuación del MEC de Lobo es orientativa

**ANEXO 5. Hoja de Consentimiento Informado**

|  |  | | | |  | |  | |  |  | | |  |  | |
| --- | --- | --- | --- | --- | --- | --- | --- | --- | --- | --- | --- | --- | --- | --- | --- |
|  | | |  | | |  | |  | |  | |  | |  |  |
|  | |  | | | | | |  | | | | | |  | |
| |  | | --- |   El paciente …………………………………………………………………………………………………………………………………  (Nombre completo del paciente)  -He leído la hoja de información que se me ha entregado  -He podido hacer preguntas sobre el estudio  -He recibido suficiente información sobre el estudio  He hablado con …………………………………………………………………….  Comprendo que la participación es voluntaria.  Comprendo que puedo retirarme del estudio:  -cuando quiera  -sin tener que dar explicaciones  -sin que ello repercuta en los cuidados médicos ni asistenciales ni de ningún otro tipo  Por todo ello presto libremente mi conformidad para participar en el estudio.  Firma del paciente Firma del investigador  …./…./…. . …./…./…. | |  | | | | | |  | | | | | |  | |
|  | | | |  | | | | | | |  | | | | |
|  | |  | | | | | |  | | | | | |  | |
| **ANEXO 6. Hoja de Información al paciente** | |  | | | | | |  | | | | | |  | |
| | **Hoja de Información al paciente**  Se le ha invitado a participar en un estudio sobre la evolución del Deterioro Cognitivo Leve. Antes de que acepte participar en este estudio lea atentamente esta hoja y no dude en preguntar cualquier duda que tenga.  **¿CUÁL ES EL OBJETIVO DE ESTE ESTUDIO?**  Valorar si añadir a la dieta habitual un alimento comercializado en España en cápsulas a base de Omega-3 puede ser beneficiosa para la sintomatología del Deterioro Cognitivo Leve (memoria…). Para ello se crearán dos grupos; a uno de ellos se les suministrará el producto y al otro no. A todos se le realizará los test habituales que se realizan en la residencia. Se recogerán algunos datos de su historia clínica.  **¿ESTOY OBLIGADO A PARTICIPAR?**  Su participación en este estudio es completamente voluntaria.  **¿CUÁLES SON LOS INCONVENIENTES Y LOS RIESGOS A PARTICIPAR?**  No cambiará la atención ni el tratamiento que recibe.  **¿SERÁ CONFIDENCIAL MI PARTICIPACIÓN EN ESTE ESTUDIO?**  Los datos que se recogen serán tratados con estricta confidencialidad de acuerdo con las exigencias legales sobre el tratamiento de datos de carácter personal (Ley Orgánica 15/1999 de 13 de diciembre de “Protección de Datos de Carácter Personal”).  **¿QUÉ SE HARÁ CON LOS RESULTADOS DEL ESTUDIO DE INVESTIGACIÓN?**  Estos resultados (anónimos) se difundirán en foros y revistas científicas manteniendo en todo momento la confidencialidad de todos los pacientes participantes.  Gracias por su atención. Si accede a participar en este estudio, se le entregará una copia de esta hoja de información para el paciente y una copia firmada del formulario de consentimiento. |  |  |  | | --- | --- | --- | --- | | | | |  | | | | | | |  | | | | |
