# Supplementary material for: Association between cognitive function and supplementation with omega-3 PUFAs and other nutrients in ≥ 75 years old patients: A randomized multicenter study
Source: PLoS One. 2018 Mar 26;13(3):e0193568. doi: 10.1371/journal.pone.0193568 (PMC5868762; doi:10.1371/journal.pone.0193568)
Supplement: S2 File — (DOCX) [file pone.0193568.s004.docx]

**Clinical trial protocol:**

**omega 3 supplementation AND EVOLUTION of Cognitive impairment in elderly**

**Type of study: Multicenter, Observational, LONGITUDINAL RANDOMIZED controlled with PLACEBO**

Dr. Joaquín Baleztena Gurrea

Geriatric Center "AMA Argaray"

Pamplona

###### INDEX

1. SUMMARY ................................................................................................... .. .. 1
2. STUDY JUSTIFICATION ....................................................................................... 3
3. Potential utility. ................................................................................................6
4. OBJECTIVES .................................................................................................... ..7
5. STUDY DESIGN ............................................................................................... .. 8
6. Statistical analysis. ......................................................................................... 13
7. ETHICAL ASPECTS……………………………………………………………….………………………. 13
8. ORGANIZATIONAL ASPECTS ......................................................................... .. 14
9. FINAL REPORT .............................................................................................. .. 15
10. APPENDICES .................................................................................................... 16

**SUMMARY**

- 1. **. Type of study**

Multicenter, observational, longitudinal, randomized vs. control group (placebo).

Pilot study

**1.2. Identification of the promoter and address**

Dr. Joaquin Baleztena Gurrea

Geriatrician

Geriatric Center "Amma Argaray"

C / Valley Egües 1

21003 PAMPLONA

**1.3. Title**

Omega 3 supplementation and evolution of cognitive impairment in elderly

**1.4. Centers where the study is planned**

Centers belonging to the institution, "Amma Navarra"

1. Geriatric Center Amma Argaray

2. Geriatric Center Amma Oblatas

3 Geriatric Center Amma Mutilva

PAMPLONA

**1.5. Principal investigator of the study**

Dr. Joaquin Baleztena Gurrea

Geriatrician

Geriatric Center "Amma Argaray"

C / Valley Egües 1

21003 PAMPLONA

**1.6. Ethics Committee**

Healthcare Ethics Committee and Clinical Research (HECCR) Geriatric Center "Amma Argaray"

# **1.7. Principal objective**

To study whether administration of a dietary supplement with omega-3 fatty acids, EPA, folic acid, vitamin B12, vitamin E and Gingko biloba [Reference (Annex 1)] produce better cognitive performance in elderly subjects without deficit or a mild cognitive deficit.

**1.8. Study disease**

Elderly patients, without cognitive impairment or mild impairment.

**1.9. Study population and sample size**

Subjects without cognitive impairment or mild impairment residents in three geriatric centers of Amma Navarra group. In this pilot study it is aimed to include a total of 170 subjects (150 + aprox. 10%), 85 supplemented with the reference product and 85 with placebo.

Crook TH, Bartus RT, Ferris SH, P Whitehouse, GD Cohen, Gershon S. Age Associated Memory Impairment: Proposed diagnostic criteria of clinical change Measures. NIMH report of a work group. Developmental Neuropsychology 1986; 2: 261-276.

**Inclusion criteria**

- Patients of age or over 80 years.
- Patients without cognitive impairment or mild impairment, selected by cognitive scales, and taking into account the report of each participant, their families and the overall judgment of the examiner. The criterion will be: Global Deterioration Scale (GDS) of Reisberg lower than 4 (GDS 1,2 and 3)
- Participants have to accept to enter to the study and sign the informed consent.
- Those subjects with systemic diseases will be included if it is judged that the disease is controlled and it will not cause any cognitive impairment during study. Sensory disturbances (vision and hearing) will be included if it is judged that they are not serious and therefore do not compromise the results.

**Exclusion criteria**

- Educational level that avoids the understanding, implementation or evaluation of the tests.
- Neurological diseases, other systemic abnormalities or poorly controlled mental disorders. This data will be obtained from medical records.
- Negative assessment by the researcher based on previous usual tests.
- Diagnosis of dementia.
- Subjects that, throughout the study suffer a permanently decline in functional performance in GDS Reisberg scale, due to illness or acute life event.
- Subjects with a history of epilepsy, seizures or convulsions.

###### **JUSTIFICATION OF STUDY**

Mild Cognitive Impairment (MCI) is a condition that its difficult to diagnose, which lies between normal aging to mild dementia and includes the risk of future dementia. It is known that approximately 60-65% of people with MCI will develop dementia throughout their life. For some authors progression since the MCI appear until the onset of dementia is about 18 months (Busse et al., 2006).

The prevalence of MCI is high in the elderly population and this data depends on several factors, being the diagnostic scale used one of the most important. According to 2 studies published in Spain the prevalence was 19-23% in 1997 and in 2002 was 13.8-19,9%. (Bermejo et al., 1997 and 2002)

Based on the results of Fisher et al. (2007) it is known that 10-15% of patients with MCI evolve Alzheimer's disease in the next year while only 1-2% of healthy subjects. A recent study assesses that 19% of patients with MCI will be diagnosed with Alzheimer's disease in the next 3 years (Okonkowo et al., 2008).

Meanwhile Fisher (2007) estimates that 46% of MCI will degenerates in Alzheimer's disease in the next 30 months. In the other hand, Salloway (2006) gives an average progression of 16% per year.

There is a divergence between studies when measured prevalence or incidence of the disease, aspect that can be linked in part to the test used (Petersen et al., 2001).

The MCI is a syndrome manifested by cognitive deficits and no or minimal interference with daily activity, not meeting criteria for dementia. However, this definition is now complemented with physiological, pathological and presence or absence of criteria of amnestic cognitive deficits (Frutos-Joy et al., 2007).

In order to detect early dementia and assess the evolution there has been developed various tests and measurements that are emerging, however, none of them is individually applied as a diagnostic tool (Boustani et al., 2003).

Tests are usually sensitive for diagnosis when the scores are low, whereas when the scores are high and close to the maximum value of test, variability can make a diagnostic accuracy difficult. This is the case of diagnosing MCI.

The MMSE is undoubtedly the most validated and used test but has problems in terms of reliability depending on the age of people and their cultural level, so it was proposed an adaptation table that offers the possibility of cutoffs (Crum was raised, 1993). There is validated version for Spanish population with the name of Mini mental Cognitive Scale (MMCS) conducted by Lobo (Lobo 1979, 1999).

It has been discussed the unreliability of the MMSE in detecting early stages of Alzheimer mainly due to the use of very simple items to assess memory and language. However, it is considered as the test of choice for longitudinal monitoring of patients (Sarasola et al., 2004).

The assessment of functional disability is an essential part of the diagnostic process in patients with dementia. Scales that best reflect the deficits are the Clinical Dementia Rating (CDR) and Global Deterioration Scale (GDS) of Reisberg. GDS provides seven possible stages from normal to severe deterioration. (Antúnez, 2005)

Regarding the MCI new studies are emerging concluding that intake of polyunsaturated fatty acids omega-3 are linked to reduce cognitive impairment. Consumption of fish rich in omega-3 (DHA and EPA) appears to decrease the risk of cognitive impairment and incident dementia (Cochrane, 2006), some studies have shown that consumption of polyunsaturated fatty acids omega-3 may delay or prevent Alzheimer's disease.

In this sense, Morris et al., between 1993 and 2000, in order to assess whether the intake of different types of omega-3 decreased the risk of developing Alzheimer's disease, conducted a prospective population based study including 815 subjects aged between 65 and 94 years who did not have Alzheimer's disease, which were evaluated over an average period of 3.9 years. They found that the group of subjects who had consumed polyunsaturated omega-3 (DHA, EPA and alpha-linoleic) fatty acids had a 60% lower risk of developing Alzheimer's disease (Morris et al., 2003).

Similarly, Freund-Levi et al., in 2006, they sought to determine whether a diet supplemented with omega-3 administered over a year, changed the evolution of Alzheimer's disease in phases mild to moderate. To do this, they selected and followed over one year a random sample of 174 patients diagnosed with mild to moderate Alzheimer's disease, which were allocated, either with a diet supplementation with omega-3 (1.7 grams of DHA and 0.6 grams of eicosapentaenoic acid) or placebo. Only one group of patients received placebo for 6 months as from the sixth month both groups received the same supplement of omega-3 fatty acids for another 6 months. At 6 months the first cognitive control was conducted and only significant differences were observed in the subgroup of patients with Alzheimer's disease with very mild cognitive impairment (MMSE> 27 points), When all patients began receiving omega-3 fatty acids, at month 3 and month 6, the cognitive impairment progression stops in all participants.

Kotani et al., in 2006 selected 39 patients (21 with mild cognitive impairment, 10 with organic brain damage and 8 with Alzheimer's disease). They received 240 mg DHA and 40 mg of ARA. After 90 days of treatment, patients with MCI who were treated showed immediate improvements in memory and attention. The organic brain damage group showed improvement in the immediate and late memory.

A more recent study, published in Archives of Neurology, that included 899 subjects without dementia, this subjects were a subpopulation of the Framingham study, and they were followed-up during 9 years. The results showed that after measuring the amount DHA in cell membranes, it was observed that those subjects that were in highest quartile of fatty acid omega-3 in the cell membrane had a significantly lower incidence of dementia (Schaefer et al., 2006).

Several studies promote the use of DHA for preventing Alzheimer's disease (Conquer 2000, Tully 2003; Barberger-Gateau 2002; Huang 2005).

Regardless of the interest involved in obtaining data on the effect of omega-3 in the Spanish population, all published studies until now, include a large ranges of age. Although it is rare to find initial cognitive impairments in patients of 80 or more years it is of interest in this group to assess the use of these fatty acids.

Strength of this study design, additionally to having a control group, is the possibility of using a fairly homogeneous population, especially to avoid biases such as variations in food intake or compliance of the daily intake of the food supplement.

Therefore, this study has been proposed to assess the effects on cognitive areas of elderly subjects with a supplementation with polyunsaturated fatty acids omega-3 in patients with MCI. The compound used in this study has as a major component of 340mg DHA and 40mg EPA. Small amounts of, phosphatidylserine, folic acid, vitamin B12, vitamin E and Gingko biloba. This study focuses on the effect of DHA because DHA has been associated in previous studies with lower risk of developing Alzheimer's disease.

For this study we will use commonly used questionnaires in assessing these patients: MMCS Lobo 35 points, Verbal Fluency, Clock Test, SPMSQ Pfeiffer and Reisberg GDS.

*REFERENCES*

Aguado C, Martínez J, Onís MC et al. Adaptación y validación al castellano de la versión abreviada de la “Geriatric Depresión Scale” (GDS) de Yesavage. Atención Primaria, 26, supl 1, 328, 2000.

1. Barberger-Gateau P, Letenneur L, Deschamps V, Pérès K, Dartigues JF, Renaud S. Fish, meat, and risk of dementia: cohort study. BMJ, 325, 7370, 932-3, 2002.
2. Bermejo FP, Gabriel RS, Vega SQ, Morales JM, Rocca WA, Anderson DW. Problems and issues with door to door, two phases surveys: An iIlustration from central Spain. Neuroepidemiology, 20:225-231, 2001.
3. Bermejo FP, Portera A, Gabriel RS et al. The prevalence of dementia and cognitive impairment in three sites in central Spain. A door-to-door Study in the Elderly. Neuroepidemiology, 16:7, 1997.
4. Boustani M, Peterson b, Hanson L, Harris R, Lohr: Screening for dementia in primary care: A summary of the evidence for the U.S. preventive Pervices Task Forse. Annals of Internal Medicine, 138, 927-93, 2003.
5. [Busse A, Angermeyer MC, Riedel-Heller SG.](../../../../pubmed/17077428%3fordinalpos=14&itool=EntrezSystem2.PEntrez.Pubmed.Pubmed_ResultsPanel.Pubmed_DefaultReportPanel.Pubmed_RVDocSum) Progression of mild cognitive impairment to dementia: a challenge to current thinking. Br J Psychiatry, 189, 399-404, 2006.
6. Conquer JA, Tierney MC, Zecevic J, Bettger WJ, Fisher RH. Fatty acid analysis of blood plasma of patients with Alzheimer´s disease, other types of dementia. Lipids, 35, 12, 1305-1312, 2000.
7. Crum RM, Anthony J.C, Bassett SS, Folstein MF. Population-based norms for the Mini-Mental State Examination by age and educational level. Journal of the American Medical Associatione 269, 18, 2386-239, 1993.
8. Fisher P, Jungwirth S,Zehetmayer, Weissgram S et al. Conversion from subtypes of mild cognitive impairment to Alzheimer dementia. Neurology, 68, 288-291, 2007.
9. Folstein M, Folstein S, McHugh P. Mini-Mental State. A practical method for grading the cognitive state of patients for the clinical. Journal of Psychiatric Research, 12, 189-198, 1975.
10. Freund-Levi Y, Eriksdotter-Jönhagen M, Cederholm T, Basun H, Faxen-Irving G. Ω-3 Fatty acid Treatment in 174 patients with mild to moderate Alzheimer Disease: Omega AD study. Arch Neurol, 63, 10, 1402-8, 2006.
11. Frutos-Alegría, M.T, Moltó Jordà JM, Morera-Guitart J, Sánchez-Pérez A, Ferrer-Navajas M. Perfil Neuropsicológico del deterioro cognitivo leve con afectación de múltiples áreas cognitivas. Importancia de la amnesia en la distinción de dos subtipos de pacientes. Rev Neurología 44, 8, 455-459, 2007.
12. Huang TL, Zandi PP, Tucker AL, et al., Benefits of fatty fish on dementia risk are stronger for those without APOE epsilon4. Neurology, 65, 9, 1409-14, 2005.
13. Kotani S, Sakaguchi E, Warashina S, Matsukawa N, Ishikura Y, Kiso Y, Sakakibara M. Dietary supplementation of arachidonic and docosahexaenoic acids improves cognitive dysfunction. Neurosci Res, 56, 2, 159-64, 2006.
14. Lobo A. Ezquerra J, Sala F. Seva J.M. Mini Examen Cognitivo; un test sencillo, práctico para detectar alteraciones intelectivas en pacientes médicos. Actas Luso Esp Neurol Psiquiatr Cienc Afines, 3, 189-202. 1979.
15. Lobo A. Saza P. Marcos G, Díaz J, De la Cámara C, Ventura T et al: Revalidación y normalización del Mini-Examen Cogniscitivo (primera versión en castellano del Mini-Mental Status Examination) en la población general geriátrica. Med Clin (Barc), 112, 767-74, 1999.
16. Morris MC, Evans DA, Bienias JL, Tangney CC, Bennett DA, Wilson RS, Aggarwal N. Consumption of fish and n-3 fatty acids and risk of incident Alzheimer Disease. Ach Neurol, 60, 940-946, 2003.
17. Okonkowo O.C, Griffith H.R, Copeland J.N:, Belue K., Lanza S. et al. Medical decision making capacity in mild cognitive impairment. Omega-3 year longitudinal study. Neurology, 71, Nov 4; 1474-1480, 2008.
18. Petersen R.C, Doody R, Kurz A, Mohs R.C. Morris JC et al. Current concepts in mild cognitive impairment. Archives of Neurology , 58, 1985-1992, 2001.
19. Salloway S. Buying Time: Management of Mild Cognitive Impairment and Early Dementia. International Psichogeriatrics, 18 (sup 1): 17-23, 2006 - resumen comité científico SIIC-.
20. Sarasola D., De Luján M., Sabe L., Caballero A., Manes F. Utilidad del *Addenbrooke’s Cognitive* *Examination* en Español para el Diagnóstico de Demencia y para la diferenciación entre Enfermedad de 18. Alheimer y la Demencia Frontotemporal. Rev. Arg. De Neuropsicol, 4, 1-11, 2004.
21. Schaefer EJ, Bongard V, Beiser AS, Lamon-Fava S, Robins SJ, Au R, Tucker KL, Kyle DJ, Wilson PW, Wolf PA. Plasma phosphatidylcholine docosahexaenoic acid content and risk of dementia and Alzheimer disease: the Framingham Heart Study. Arch Neurol, 63, 11, 1545-50, 2006.

Sheikh JI, Yesavage JA. Geriatric Depression Scale (GDS): Recent evidence and development of a shorter version. En: Brink TL eds. Clinical Gerontology: A guide to assessment and intervention. New York: Haworth Press, 165-173, 1986.

1. Solomon PR, Hirschoff A, Kelly B, Relin M, Brush M, de Veaux MD et al. A 7 minute neurocognitive screen battery highly sensitive to Alzheimer’s disease. Archives of Neurology, 55, 349-355, 1998.
2. Tully AM, Roche HM, Doyle R, Fallon C, Bruce I, Lawlor B, Coakley D, Gibney MJ. Low serum cholesteryl ester-docosahexaenoic acid levels in Alzheimer´s disease: a case-control study. British Journal of Nutrition, 89, 4, 483-489, 2003.
3. Vinyoles E., Vila J, Argimon J.M. et al. Concordancia entre el Mini-Examen Cognoscitivo y el Mini-Mental State Examination en el cribado del déficit cognitivo. Aten Primaria, 30, 1, 5-15, 2002.

Wechsler, D. WMS-III. Escala de Memoria de Wechsler III. Madrid: TEA Ediciones; 2004.

Yesavage JA, Brink TL, Rose TL. Development and validation of a geriatric depression screening scale: a preliminary report. J Psychiatry Res, 17, 37-49, 1983.

**3. POTENTIAL APPLICATIONS**

As seen above, several epidemiological studies have confirmed that consumption of omega-3 is associated with a reduced risk of cognitive impairment. It seems that higher concentrations of DHA + EPA of the normal in the general population produces a protective effect against cognitive impairment type.

It has been studied the possible physiological pathways of omega-3 acids and its benefits over MCI: neuroprotective metabolites, reducing arachidonic acid metabolites and increase of certain neurotrophic factors. Some of these pathways would also have the effect of reducing the risk of cardiovascular disease.

A clear knowledge of the effect of the supplement on the cognitive areas of elderly subjects with impaired memory would allow us to introduce flattering dietary supplements or specific preventive aspects for these people with the aim of:

1. Implementing preventive activities in patients with memory impairment due to age giving supplementation with omega-3 fatty acids.
2. Delay the onset of cognitive impairment in these subjects.

***REFERENCES***

- Morris MC, Evans DA, Tangney CH et al. Fish consumption and cognitive decline with age in a large community study. Arch Neurol, 62, 1849-1853, 2005.
- Cole GM et al. Omega 3 fatty acids and dementia. doi: 10.1016/j.plefa.2009.05.015
- Lim WS, Gammack JK et al. Ácidos grasos omega 3 para la prevención de la demencia (revisión Cochrane traducida). Biblioteca Cochrane Plus, 2007, 3 Chichester UK. John Wiley and sons Ltd
- Kotani S., Sakaguchi E., Warashina S., et al. Dietary supplementation of arachidonic and docosahexaenoic acids improves cognitive disfunction. Neuroscience research 56 (2006) 159-164.
- Petersen, R.C., 2004. Mild cognitive impairment as a diagnostic entity. Journal of Internal Medicine Sep;256, 183-194.
- Winblad B., Palmer K., Kivipelto M., Jelic V., . Fratiglioni. Mild cognitive impairment – beyond controversies, towards a consensus: report of the International Working Group on Mild Cognitive Impairment. Journal of Internal Medicine 2004; 256: 240–246
- Freund-Levi, M. Eriksdotter-Jo¨nhagen, T. Cederholm, H. Basun, G. Faxe´n-Irving, A. Garlind, Inger Vedin, MSci; Bengt Vessby, Lars-Olof Wahlund, J. Palmblad, W-3 Fatty Acid Treatment in 174 Patients With Mildto Moderate Alzheimer Disease: OmegAD Study.A Randomized Double-blind Trial ARCH Neurology 2006 vol 63, 1403-1408
- Whalley, H. C Fox, K. W Wahle, J. M Starr, I J Deary. Cognitive aging, childhood intelligence, and the use of food supplements: possible involvement of n_3 fatty acids1–3. Am J Clin Nutr 2004;80:1650 –1657.
- Kalmijn, M. P.J. van Boxtel, M. Ocké, W. M. M. Verschuren, D. Kromhout, L.J. Launer. Dietary intake of fatty acids and fish in relation to cognitive performance at middle age. Neurology 2004; 62:275-280
- Solfrizzi, C. Capurso, A. D’Introno, A.M. Colacicco, V. Frisardi,A. Santamato, M. Ranieri, P. Fiore, G. Vendemiale, D. Seripa, A. Pilotto,A. Capurso, F. Panza. Dietary fatty acids, age-related cognitive decline,and mild cognitive impairment The Journal of Nutrition, Health & Aging, 12, 6, 382-386, 2008.
- Schaefer, V. Bongard, A. S. Beiser, S. Lamon-Fava, S. J. Robins, R. Au, K. L. Tucker, D. J. Kyle, P. W. F. Wilson, P. A. Wolf. El contenido plasmático de ácido docosahexanoico en fosfatidilcolina y el riesgo de demencia y enfermedad de Alzheimer. Estudio cardiovascular de Framingham***.*** Arch neurology, 2006, 63, 1545-1550
- Fontani, F: Corradeschi, A: Felici, F. Alfatti, S. Migliorini, L.Lodi. Cognitive and physiological effects of Omega-3 polyunsaturated fatty acid supplementation in healthy subjects. European Journal of Clinical Investigation, 2005, 35, 691-699.
- Cole, Q. Ma, S.A. Frautschy. Ácidos grasos omega-3 y Demencia.Official Journal of the International Society for the Study of Fatty Acids and Lipid. (2009), doi:1 0.1016 /j. plefa.2009. 05.015.
- Yurko-Mauro, Deanna McCarthy, Dror Rom, Edward B. Nelson, Alan S. Ryan, Andrew Blackwell, Norman Salem, Mary Stedman. Beneficial effects of docosahexaenoic acid on cognition in age-related cognitive decline Alzheimer's & Dementia: The Journal of the Alzheimer's Association DOI: 10.1016/j.jalz.2010.01.013

#### **4. OBJECTIVES**

**4.1. Principal objective**

Study whether the administration of a dietary supplement with omega-3 and other substances contained in it, over one year, produce better cognitive performance in elderly subjects without deficits or mild cognitive deficits.

To do this we will describe the changes in the following questionnaires: Minimental cognitive scale of Lobo 35 points, Verbal Fluency, Clock Drawing Test, Reisberg GDS and SPSMQ Pfeiffer.

# **4.2. Secondary objectives**

- Study the characteristics of subjects who achieve better results with the supplement differentiating those who do not obtain benefit.
- Consider whether there are changes in other cognitive areas: memory, executive functions, attention, language speed processing.

**5. STUDY DESIGN**

**5.1. Summary**

Multicenter, observational, longitudinal, randomized, controlled with placebo study.

One group will receive 3 capsules of the supplement of omega-3 fatty acids of the reference product. Total: 1.050mg of DHA and 120 mg EPA. The control group will receive placebo.

Participants randomly will be assigned to received "supplement" or "placebo".

Subjects of the geriatric centers will be selected based on the inclusion and exclusion criteria using GDS scale of Reisberg (Stages 1, 2 and 3) and will be included in the protocol. They will be excluded from the study if they do not follow the treatments.

# **5.2. Methodology**

Without altering the normal practice or the existing protocol for these patients in the Geriatric Center, they will apply the questionnaires commonly used.

Patients without cognitive impairment or mild impairment will be included. It is intended to have at the end of the study a minimum of 150 patients (recruit 170) total evaluable patients (75 for the dietary supplement -include 85-, and 75 for the placebo –include85-).

A first interview with the doctors and psychologists will be conducted. All staff will be trained on administered tests to ensure inter-rater reliability. The assessment also includes a detailed medical history.

The assignment to the control or experimental group will be in time that the patient is included in the study by randomization using a computer program and personal data will dissociate through separate sheet (ANNEX 2)

All candidates will be informed of the study by the professional and will be asked to sign an informed consent at Visit 0.

The follow-up time will be 1 year (Visit 0) counting from the time the patient starts taking the supplement or placebo. Neuropsychological and mood assessment will be conducted at baseline (Visit 0), at 6 months (Visit 1) and after 1 year (Visit 2).

The manufacturer laboratory will provide the food supplement.

One group will take 3 capsules of omega-3 supplement marketed in Spain: minimum total of 75 patients -include 85-. Another similar group is the control group to which a placebo will be administered.

Being hospitalized patients, after the visit 0, a capsule will be included in their diet (the intervention group). One dietary supplement capsule at breakfast, lunch and dinner. Total: 3capsules; therefore we need 1080 capsules per patient / year of study. The control group will be given placebo.

**The clinical evolution of patients will be assessed using different scales:**

- **Global deterioration scale (GDS)**. Provides 7 possible stages: 1 = normal, 2 = very slight impairment, 3 = mild impairment, 4 = moderate damage, 5 = moderate-severe deterioration, 6 = severe impairment and 7 = very severe deterioration. It describes each stage in operational terms and based on a supposedly homogeneous deterioration. It is one of the most complete, simple and useful scales for estimating the severity of dementia.
- **Mini Mental State Examination** (MMSE) of Folstein et al., a validated version to Spainish by Lobo et al. It is based on a questionnaire widely used to explore the following areas: temporal orientation, spatial orientation, fixation, memory, naming, repetition, comprehension, reading, writing and drawing.

Folstein, MF., Folstein, SE., McHugh, PR. Mini Mental State: A practice method for grading the cognitive state of patients for the clinician. Journal of Psychiatric Research, 12, 189-198, 1975.

Lobo A. Ezquerra J, Sala F. Seva J.M. Mini Examen Cognitivo; un test sencillo, práctico para detectar alteraciones intelectivas en pacientes médicos. Actas Luso Esp Neurol Psiquiatr Cienc Afines, 3, 189-202. 1979.

Lobo A. Saza P. Marcos G, Díaz J, De la Cámara C, Ventura T et al: Revalidación y normalización del Mini-Examen Cogniscitivo (primera versión en castellano del Mini-Mental Status Examination) en la población general geriátrica. Med Clin (Barc), 112, 767-74, 1999

- Questionnaire of functional activity of Pfeiffer (SPMSQ "), which evaluates the degree of autonomy to perform daily tasks such as managing money, shopping, making food, understand and discuss news, go out alone, etc. .

Pfeffer RI, TT Kurosaki, Harrah CH, Chance JM, Bates D, Detels R et al. A survey tool for senile dementia diagnosis. Am J Epidemiol 1981; 114: 515-7.

- **Verbal fluency.** The semantic verbal fluency test, despite its simplicity and easy application, it has been very sensitive to diagnose brain damage and especially cognitive impairment, alone or as part of various neuropsychological tools.

Rosen W .: "Verbal fluency in aging and dementia". J. Clin Neuropsychol 2: 135-146, 1980

- **Clock Test.** The Drawing Clock Test is a simple test, for early detection, to evaluate the cognitive status of the subject, and it is used in both clinical practice and research. This test evaluates different mechanisms involved in the execution and planning of motor activities, mainly visual-perceptives functions, visual-motor and visual-constructive.

Battersby, WS, Bender, MB, Pollack, M. Kahn, RL (1956): Unilateral "spatial agnosia" ( "inattention") In Patients With cortical lesions. Brain, 79: 68-93.

Process scheme:

|  | **Day O**  **(Visit 0)** | **Month 6**  **(Visit 1)** | **Month 12**  **(Visit 2)** |
| --- | --- | --- | --- |
| **Informed consent** | **X** |  |  |
| **GDS** | **X** |  | **X** |
| **MMCS** | **X** | **X** | **X** |
| **Functional activity Pfeiffer** | **X** |  | **X** |
| **Verbal frequency** | **X** |  | **X** |
| **Clock Test** | **X** |  | **X** |

Data for each visit (Visit 0, visit 1 and visit 2) are collected in the corresponding Data Collection Sheet (Annex 3)

**5.3. Variables**

- Demographic (age, sex, education)
- Diseases. Presence or absence of:

thyroid disorders

diagnosis of cardiovascular or cerebrovascular disease.

Cardiovascular risk factors: hypertension, diabetes, obesity, smoking, dyslipidemia

- Depression (anamnesis on its presence will be performed)
- Chronic use of psychoactive drugs
- Participation in cognitively stimulating activities

| Variable | Definition | Scale |
| --- | --- | --- |
| Age | Patient age at the time of beginning of study | In years |
| Sex |  | Man / woman |
| Studies | Educational level | Illiterate  functional illiterate  primary  Bachelor  Media  university |
|  | Years of study | In years |
| former habitual occupation |  | Business Management  Technical / Professional  Commerce  qualified worker  Unskilled worker  Housewife |
| Diseases | Presence or absence of:  thyroid disorders  previous diagnosis of cardiovascular or cerebrovascular disease.  Cardiovascular risk factors: hypertension, diabetes, obesity, smoking, dyslipidemia | IF NOT |
| Mood disorder | Depression | IF NOT |
| Consumption of drugs | Psychoactive drugs taken chronically by type: Benzodiazepines or anxiolytics, hypnotics, antidepressants, neuroleptics, other psychotropic drugs | IF NOT |
| GDS | Cognitive impairment scale | 1-7 |
| MMCS | General cognitive performance | 0-35 |
| Pfeiffer functional activity questionnaire | Current memory | More than 5 = problem |
| Verbal fluency | Executive functions | <13 in 1 min = problem |
| Clock test | Praxia, visoconstruction | 0-10 |

**5.2.1 Previous Determinations**

Following the usual protocol in dementia units, patients have their own medical record information prior to inclusion. These data will be taken into account in assessing the exclusion criteria.

**5.2.2. Supplying the supplement**

As they are hospitalized patients, in the intervention group, the supplement will be provided in each of the main meals (3 daily) a total of 1080 capsules will be needed during the study period, and they will be delivered at a rate of 90 capsules / patient / month . The control group will be given placebo.

# **5.3. Variables**

For each patients we will used the following scales: Global Deterioration Scale (GDS) of Reisberg, MMCS Lobo, Pfeiffer Questionnaire, Verbal Frequency and Clock Test.

**5.4. Definition of the study population: selection criteria**

**5.4.1. Inclusion criteria**

- Patients of 80 years or older.
- Patients without cognitive impairment or mild impairment, selected by cognitive scales and taking into account the report of the person, their relatives and the overall judgment of the examiner. Criterion: Global Deterioration Scale of Reisberg lower than 4 (GDS 1,2 and 3)
- Accept entering the study and sign the informed consent.
- Subjects with systemic diseases will be included if it is judged that are controlled and that the disease does not occur during cognitive impairment study in the subject. Sensory disturbances (vision and hearing) will be included if it is judged that they are not serious and therefore do not compromise the results.

**5.4.2. Exclusion criteria**

- Cultural level that do not allow understanding or implementing the test.
- Having neurological diseases poorly controlled mental disorders or any other systemic abnormalities. This data will be obtained from medical records.
- Negative assessment by the researcher.
- Diagnosis of dementia.
- Subjects that during the study suffer a sharp permanently decline in functional performance (2 stages in Reisberg GDS scale), due to illness or acute life event.
- Subjects with a history of epilepsy, seizures

#### **5.5. Observational period**

The duration of data collection will be 12 months, or from baseline to final visit 0 or Visit 2 with assessment at 6 months (Visit 1).

#### **5.6. Sample size**

There are numerous discrepancies regarding the natural evolution of annually cognitive impairment. This depends on the used questionnaire and age.

If we accept that 20% of the people over 80 years with cognitive impairment present natural progression and evolution, and with this intervention may be only 5%, with a 15% improvement intergroup and assume an α risk = 0.05, and a β risk = 0.20, the sample size would be 75 patients per group. Total: 150 to evaluate. Presuming losses of around 10%, we should include 170 patients for the intention to treat analysis, two groups of 85. The calculation was conducted with software EPICAL 2000.

**6. Statistical Analysis**

To study the differences between control and intervention groups ANOVA, general linear model with repeated measures, or mean difference will conducted. It will be controlled by age, sex, depression and other descriptive variables that are considered appropriate. The intra-subject factor will be the time (1st evaluation cut at six months and 12 months) and the between-subjects factor is the group that has been assigned (control or intervention).

The relative risk will be calculated. The effect size will be studied with ETA statistic.

To study the predictors of improvement we will perform a logistic regression model; the effect size will be calculated with the odds ratio using the software IBM SPSS 19.0.

**7. ETHICAL ASPECTS**

The researcher must conduct the study according to the principles of the Declaration of Helsinki related to medical research in humans. Copies can be obtained through the website (World Medical Association) World Medical Association<http://www.wma.net/e/policy/b3.htm>.

The study should develop according to the protocol ensuring compliance with the standards of Good Clinical Practice.

**7.1. Risk-benefit assessment for research subjects**

The development of the study does not involve additional risks to the patient, except for proper management of their pathology in routine clinical practice.

**7.2. Information sheet and Informed Consent form**

In compliance with the Declaration of Helsinki, it is the responsibility of the researcher / contributor to inform the patient about his involvement in this project. Participation is voluntary and does not involve any change in either treatment or medical care compared to those who receive in case he didnot participate. The patient will receive appropriate information and an informed consent will be obtained before reviewing their medical history and before being included in the study (Annex 4 and Annex 5).

**7.3. Data confidentiality**

The personal data of the participants will be disassociated data. It will also follow the provisions of Law 15/1999 of 13 December on "Protection of Personal Data" for handling unlinked personal data.

In the data collection form the patient will be identified only by a code. The researcher will keep a confidential record that relates identification codes with patient identification (Annex 2)

**7.4. Interference with the medical prescription habits**

This study develops within normal clinical practice only being included a food supplement based of polyunsaturated fatty acids omega-3 in the intervention group and placebo in the control group.

**8. ORGANIZATIONAL ASPECTS**

1. Preparation and presentation of the thesis project
2. Presentation of the project and protocol to the Ethics Committee
3. Progressive collection of subjects meeting the criteria of inclusion and exclusion
4. Information to patients and written informed consent
5. Randomization of subjects: Control / experimental.
6. Start with the food supplementation and placebo
7. Neuropsychological and behavioral ratings

(V0, V1 and V2) entered in the computer data

1. Data analysis. Hypothesis testing.
2. Preparation of final results
3. Elaboration of thesis and possible publication

**9. FINAL REPORT**

The final report of results will take place in a maximum of six months after the end of the observational study.

**9.1. Communication of research findings**

The results will be used for obtaining the degree of PhD. and they will be communicated through scientific publications, specialized conferences or other specific way. The communication of the clinical study reports will be independent from the results.

**10. ATTACHMENTS**

ANNEX 1: Description of nutritional supplement containing omega-3

ANESO 2: Dissociation Data Sheet

ANNEX 3: Data Collection Sheet

ANNEX 4: Test and scales used

ANNEX 5: Informed Consent

ANNEX 6: Patient Information Sheet

**ANNEX 1. Supplement omega-3 (ACUTIL)**

## Food supplement containing polyunsaturated fatty acids omega-3 Ginkgo biloba, phosphatidylserine, vitamin E, vitamin B12 and folic acid.

Fish oil, a natural source of polyunsaturated fatty acids omega-3 (EPA and DHA), associated with a balanced diet, can positively influence in the reduction of low density lipoprotein (LDL) which can improve both cardiovascular and cognitive function.

Tree leaves of Ginkgo biloba contain substances whose main effects appear to be related to its antioxidant properties, contributing to the cellular mechanisms against oxidation.

Phosphatidylserine, it belongs to a class of chemical compounds known as phospholipids. Phosphatidylserine is involved in maintaining the structural integrity of the cell membrane.

Vitamin E, which is at level of cell membranes, helps to regulate many metabolic reactions.

Vitamin B12 is a water soluble vitamin, which promotes the formation of blood cells and neurons.

Folic acid, a vitamin B complex are involved in methylation processes that are essential for neuronal development.

**Ingredients**:

Fish oil polyunsaturated fatty acids omega-3; gelatin capsule (coating agent: gelatin humectants: glycerol, sorbitol; dye: E172); standardized extract of Ginkgo biloba leaves (6%); emulsifier: glyceryl monostearate; phosphatidylserine *; D-alpha tocopherol; cyanocobalamin; teroilmonoglutámico acid.

* From soy.

| NUTRITIONAL INFORMATION | Per daily dose  (1 capsule) | % RDA for 1 cps |
| --- | --- | --- |
| Polyunsaturated fatty acids omega-3 | 350 mg | - |
| of which EPA * - eicosapentaenoic acid | 40 mg | - |
| * DHA - docosahexaenoic acid | 250 mg | - |
| phosphatidylserine | 15 mg | - |
| Vitamin E | 5 mg α-TEA | 50 |
| B12 vitamin | 5 μg | 500 |
| Folic acid | 250 μg | 125 |

RDA: Recommended Daily Allowance

* Mean values

| VEGETABLE COMPONENTS | Per daily dose  (1 capsule) |
| --- | --- |
| Standardized extract of Ginkgo biloba leaves | 60 mg |
| providing  24% of glucosides ginkgoflavonics | 14.5 mg |
| 6% of ginkgolides and bilobalide | 3.6 mg |

How to use:

Take 1 capsule daily with food or drink. If you take this product for the first time, you can double the dose during the first 12 weeks.

**Warnings**:

Store in a cool and dry place. Keep out of the reach of children. Food supplements should not be used as substitutes for a balanced diet. Do not exceed the recommended daily dose.

#### Presentation: 30 capsules

**Made by**:

Efamol Ltd

14 The Mole Business Park

Leatherhead, Surrey

KT22 7BA - UNITED KINGDOM

**Marketed by**:

ANGELINI PHARMACEUTICAL, SA

C. Osi, 7-08034 Barcelona

**ANNEX 2. Dissociation Data Sheet**

| \| No. Dissociation \| Initials/  Health record \|  \| No. Dissociation \| Initials/  Health record \| \| --- \| --- \| --- \| --- \| --- \| \| 1 \|  \|  \| 31 \|  \| \| 2 \|  \|  \| 32 \|  \| \| 3 \|  \|  \| 33 \|  \| \| 4 \|  \|  \| 3. 4 \|  \| \| 5 \|  \|  \| 35 \|  \| \| 6 \|  \|  \| 36 \|  \| \| 7 \|  \|  \| 37 \|  \| \| 8 \|  \|  \| 38 \|  \| \| 9 \|  \|  \| 39 \|  \| \| 10 \|  \|  \| 40 \|  \| \| 11 \|  \|  \| 41 \|  \| \| 12 \|  \|  \| 42 \|  \| \| 13 \|  \|  \| 43 \|  \| \| 14 \|  \|  \| 44 \|  \| \| 15 \|  \|  \| 45 \|  \| \| 16 \|  \|  \| 46 \|  \| \| 17 \|  \|  \| 47 \|  \| \| 18 \|  \|  \| 48 \|  \| \| 19 \|  \|  \| 49 \|  \| \| 20 \|  \|  \| 50 \|  \| \| 21 \|  \|  \| 51 \|  \| \| 22 \|  \|  \| 52 \|  \| \| 23 \|  \|  \| 53 \|  \| \| 24 \|  \|  \| 54 \|  \| \| 25 \|  \|  \| 55 \|  \| \| 26 \|  \|  \| 56 \|  \| \| 27 \|  \|  \| 57 \|  \| \| 28 \|  \|  \| 58 \|  \| \| 29 \|  \|  \| 59 \|  \| \| 30 \|  \|  \| 60 \|  \| |
| --- | --- | --- | --- | --- | --- | --- | --- | --- | --- | --- | --- | --- | --- | --- | --- | --- | --- | --- | --- | --- | --- | --- | --- | --- | --- | --- | --- | --- | --- | --- | --- | --- | --- | --- | --- | --- | --- | --- | --- | --- | --- | --- | --- | --- | --- | --- | --- | --- | --- | --- | --- | --- | --- | --- | --- | --- | --- | --- | --- | --- | --- | --- | --- | --- | --- | --- | --- | --- | --- | --- | --- | --- | --- | --- | --- | --- | --- | --- | --- | --- | --- | --- | --- | --- | --- | --- | --- | --- | --- | --- | --- | --- | --- | --- | --- | --- | --- | --- | --- | --- | --- | --- | --- | --- | --- | --- | --- | --- | --- | --- | --- | --- | --- | --- | --- | --- | --- | --- | --- | --- | --- | --- | --- | --- | --- | --- | --- | --- | --- | --- | --- | --- | --- | --- | --- | --- | --- | --- | --- | --- | --- | --- | --- | --- | --- | --- | --- | --- | --- | --- | --- | --- | --- | --- | --- |

| No. Dissociation | Initials/  Health record |  | No. Dissociation | Initials/  Health record |
| --- | --- | --- | --- | --- |
| 61 |  |  | 91 |  |
| 62 |  |  | 92 |  |
| 63 |  |  | 93 |  |
| 64 |  |  | 94 |  |
| 65 |  |  | 95 |  |
| 66 |  |  | 96 |  |
| 67 |  |  | 97 |  |
| 68 |  |  | 98 |  |
| 69 |  |  | 99 |  |
| 70 |  |  | 100 |  |
| 71 |  |  | 101 |  |
| 72 |  |  | 102 |  |
| 73 |  |  | 103 |  |
| 74 |  |  | 104 |  |
| 75 |  |  | 105 |  |
| 76 |  |  | 106 |  |
| 77 |  |  | 107 |  |
| 78 |  |  | 108 |  |
| 79 |  |  | 109 |  |
| 80 |  |  | 110 |  |
| 81 |  |  | 111 |  |
| 82 |  |  | 112 |  |
| 83 |  |  | 113 |  |
| 84 |  |  | 114 |  |
| 85 |  |  | 115 |  |
| 86 |  |  | 116 |  |
| 87 |  |  | 117 |  |
| 88 |  |  | 118 |  |
| 89 |  |  | 119 |  |
| 90 |  |  | 120 |  |

| No. Dissociation | Initials/  Health record |  | No. Dissociation | Initials/  Health record |
| --- | --- | --- | --- | --- |
| 122 |  |  | 152 |  |
| 123 |  |  | 153 |  |
| 124 |  |  | 154 |  |
| 125 |  |  | 155 |  |
| 126 |  |  | 156 |  |
| 127 |  |  | 157 |  |
| 128 |  |  | 158 |  |
| 129 |  |  | 159 |  |
| 130 |  |  | 160 |  |
| 131 |  |  | 161 |  |
| 132 |  |  | 162 |  |
| 1331 |  |  | 163 |  |
| 134 |  |  | 164 |  |
| 135 |  |  | 165 |  |
| 136 |  |  | 166 |  |
| 137 |  |  | 167 |  |
| 138 |  |  | 168 |  |
| 139 |  |  | 169 |  |
| 140 |  |  | 170 |  |
| 141 |  |  |  |  |
| 142 |  |  |  |  |
| 143 |  |  |  |  |
| 144 |  |  |  |  |
| 145 |  |  |  |  |
| 146 |  |  |  |  |
| 147 |  |  |  |  |
| 148 |  |  |  |  |
| 149 |  |  |  |  |
| 150 |  |  |  |  |
| 151 |  |  |  |  |

**ANNEX 3. Data Collection Sheet**

Evaluation Date: ___ / ___ / ______

Center code Number of internal dissociation No.

**CLINICAL DATA AND HISTORY SOCIODEMOGRAPHIC**

| Date of birth | __ / __ / __ | | | | Age: | | ___ years | | | Sex: | | 1 ⬜ F | | | |  | Years schooling: _____ | | |
| --- | --- | --- | --- | --- | --- | --- | --- | --- | --- | --- | --- | --- | --- | --- | --- | --- | --- | --- | --- |
|  | | |  | | |  | | |  | | | 2 ⬜ M | | |  | |  | |  |
| Educational level | | 1 ⬜ Illiterate | | | | | | | 3 ⬜ Primary studies | | | | | | | | 5 ⬜ middle studies | | |
|  | | 2 ⬜ functional illiterate | | | | | | | 4 ⬜ elementary degree | | | | | | | | 6 ⬜ Higher education | | |
|  | | | |  | | | | | | | | | |  | | | | | |
| Profession | | 1 ⬜ Business Management | | | | | | | 3 ⬜ Commerce | | | | | | | | 5 ⬜ Unskilled worker | | |
|  | | 2 ⬜ Technical / Professional | | | | | | | 4 ⬜ qualified worker | | | | | | | | 6 ⬜ Housewife | | |
|  | | | |  | | | | | | | | | |  | | | | | |
| Personal Background | | 1 ⬜Cardiovascular disease | | | | | | | 6 ⬜ thyroid disorders | | | | | | | | 10 ⬜Psychiatric disease | | |
|  | | 2 ⬜ Hypertension | | | | | | | 7 ⬜ Parkinson disease | | | | | | | | ⬜ Depression | | |
|  | | 3 ⬜ Diabetes | | | | | | | 8 ⬜ smoking | | | | | | | | ⬜ Anxiety | | |
|  | | 4 ⬜ dyslipidemia | | | | | | | 9 ⬜ Alcoholism | | | | | | | | ⬜ other | | |
|  | | 5 ⬜ Stroke | | | | | | |  | | | | | | | |  | | |
|  | | | |  | | | | | | | | | |  | | | | | |
| Actual medication | | 1 ⬜ Benzodiacepin / anxiolytics / hypnotics | | | | | | | 5 ⬜ antiepileptic | | | | | | | |  | | |
|  | | 2 ⬜ antidepressants | | | | | | | 6 ⬜ other psychiatric drugs | | | | | | | |  | | |
|  | | 3 ⬜ neuroleptics | | | | | | |  | | | | | | | |  | | |
|  | | 4 ⬜ antiparkinsonian | | | | | | |  | | | | | | | |  | | |
|  | | | |  | | | | | | | | | |  | | | | | |
|  | |  | | | | | |  | | | | |  | | | | |  | |
|  | |  | | | | | | | | | | | |  | | | | | |
| Do you perform cognitive stimulation? | | | | | | | | 1 ⬜ YES | | | 2 ⬜ NO | | | | | | | | |

**TEST SCORES**

| **Test** | **Visit 0** | **Visit 1** | **Visit 2** |
| --- | --- | --- | --- |
| **MEC** |  |  |  |
| **Pfeffer questionnaire** |  |  |  |
| **Verbal frequency** |  |  |  |
| **Clock Test** |  |  |  |
| **Reisberg GDS** |  |  |  |

Adverse effects

**ANNEX 4. Test and scales to apply**

Evaluation Date: ___ / ___ / ______

**(MMSE)**

Patient................................................. ......................................Age........... .....

Occupation................................................. .....Scholarship....................................

Examined by ................................................ ..Date..........................................

**ORIENTATION**

• Tell me the day ...... ..month ........... ...... ....................... Season .....................Year..........

­**___5**

• Tell me the name of the hospital (or place) ........................................... ..................................

Floor ..................... City................. ......... Province .............Country................

**___5**

**FIXATION**

• Repeat these three words; peseta, horse, apple

**­­___3**

**CONCENTRATION AND CALCULATION**

• If you have 30 euros and giving me three how many are left?

**___5**

• Repeat these three numbers: 5,9,2.

**___3**

**MEMORY**

• Remember the three words I mention before?

**___3**

**LANGUAGE AND CONSTRUCTION**

• Show a pen. What is this? Repeat with a clock

**___2**

• Repeat this sentence: In a wheat field there were five dogs

**___1**

• An apple and a pear are fruits. Are they?

What are red and green?

**­­___2**

• What are a dog and a cat?

**___3**

• Take this paper with your right hand, fold it and put it on the table

**­­___1**

• Read this and do what it says: CLOSE YOUR EYES

**___1**

• Write a sentence

**­­___1**

• Copy this drawing___1

**APPLICATION OF TEST**

**Orientation:**

Follow the orders of the test (one point each correct answer).

**Fixation:**

Clearly repeat each word in a second. They are given as many points as repeated words correctly on the first try. Emphasize that they have to remember because they will be asked later.

**Concentration:**

If the patient do not understand it can rephrase the question as follows: "If you have 30 euros and gives me 3 euros How many are left? and then keep giving me 3 on 3 (without repeating the amount he gives). One point for each correct subtraction, exclusively. For example 30 least 3 = 28 (0 points); if the next subtraction is 25, it is correct (1 point).

Repeat slowly digits: 1 second each until the patient learn. After asking him to repeat them in reverse order and it is given one point for each correct digit place in reverse opposition.mFor example, 592 (295 is correct); if it says 925 corresponds to 1 point.

**Memory:**

Follow the instructions of the test, giving enough time to remember, but without helpiing (one point for each word recalled)

**Language and construction:**

Follow the instructions noting that:

- Read the phrase slowly and properly articulated. To grant 1 point it must be repeated in the first attempt and properly articulated, a failure in one letter is 0 points.
- Similarities; to give a point in green-red has to answer "colors". For dog-cat likeness exclusive right answer is animal or animals "x".
- In the verbal commands, if you take the paper with the left hand is a fault in that section. If you bent more than twice is another failure. Depending on the patient's position may modify the order to put the paper on the table or floor. Each of the parts of the order is executed correctly point to a maximum of 3.
- Test for reading and writing, ask the patient to place his glasses, (in case he use), and if its necessary write the order and the pentagons on the back of the paper, to see them perfectly. It is awarded a point if, no matter what he reads aloud, and close his eyes without insisting. Emphasized before, twice at maximum, to read and do what its written on the paper.
- To write a sentence instruct him that it has to be different from his name. If its needed you can use an example, but insisting that he has to write something different. It requires a subject, a verb and a complement to give a point (the impersonal sentences without subject)

*Mini-Examen Cognoscitivo. Versión española del Mini-Mental Status Examination de Folstein et al (1975). Validado por Lobo et al (1979-94)*

*Publicación más representativa :* un sencillo, práctico, para detectar alteraciones intelectuales en paciente médicos. Actas Luso-Españolas de Neurología, psiquiatría y ciencias afines vol 3, 189-202, 1979

**Short Portable Mental State Questionnaire (SPMSQ) Pfeiffer**

Very fast and easy to use for "screening" by the primary care physician

| **SPMSQ Pfeiffer** | |
| --- | --- |
| **Ask perform** | **Mistakes** |
| Total (maximum 10 points) |  |
| What's the date today? (Day, month and year) |  |
| What weekday is today? |  |
| Where are we now? (Place or building) |  |
| What is your phone number? (Or address if no phone) |  |
| How old are you? |  |
| When you were born? (Day, month and year) |  |
| What is the name of the Prime MInister? |  |
| Who was the former President of the Government? |  |
| What is the surname of your mother? |  |
| Subtract three to twenty and keep subtracting 3 to the amount |  |

One point is awarded for each error, For example, a single failure in the sequence of subtractions will give the wrong series and therefore will add a point. Same with dates: if the patient do not correctly predict the month, day or year, will be considered erroneous date, although he guesses the other two components.

a total of 5 or more points is considered pathological, allowing more error if the patient has not received primary education or less wrong if you have received higher education.

**VERBAL FLUENCY TEST**

**Tell me all the animals that you know.**

We have to count all animals that he say in one minute. If he say less than 13, we will suspected cognitive impairment

The cutoff point may vary according to the characteristics of the population and the sensitivity and specificity you want to achieve

Decreased spontaneity and verbal fluency difficulty can be assessed by this instrument. This test measures the number of elements of a category that the patient can evoke at a given time, usually a minute; This is a complex task from the cognitive point of view, involved in language, mnemonic and executive processes.

It is a very easy and fast application test that does not require any equipment and can be applied to subjects who can not be evaluated by other tools, (like illiterate people, sensory deficits such as blindness, motor deficits, etc.) or in situations where other tests are difficult or impossible to perform as in the case of bed-ridden patient. The VFT is very sensitive to all kinds of brain damage, affecting early in processes that determine cognitive impairment and especially in Alzheimer's Disease. The application environment of proof always dictate the optimal cutoff, depending on whether we want to minimize false negatives, population studies, screening) or false positives, use the test as a confirmatory test:

The VFT would be ideal for application in screening studies eliminating from a second phase all those subjects who score above the cut point; the result might be improved if we chose a cutoff point to improve the sensitivity (<13) at the expense of a greater number of false positives is chosen.

Likewise, it can be an excellent test to confirm the presence of dementia in subjects in which it is suspected on the basis of a previous test as the MMSE; in this regard, one can choose a cut off with greater specificity (<9), whereby false negatives would be virtually nonexistent.

**GDS Reisberg**

Global Deterioration Scale of Reisberg

**GDS-1, absence of cognitive impairment**

(Minimental cognitive scale of Lobo 30 to 35 points). It corresponds to a normal individual:

Absence of subjective complaints.

Absence of apparent memory disorders in the clinical interview.

**GDS-2, very mild cognitive decline**

(Minimental cognitive scale of Lobo 25 to 30 points). It corresponds with subjective cognitive impairment:

Subjective complaints of memory defects, especially in:

a) forgot where he has placed familiar objects.

b) Forgetting previously well-known names.

There is no objective evidence of memory defects on clinical examination.

No objective defects in work or social situations.

There is full knowledge and appreciation of symptoms.

**GDS-3, mild cognitive defect**

(Minimental cognitive scale of Lobo between 20 and 27 points). It corresponds with mild cognitive impairment:

First clear defects: demonstrations in one or more of these areas:

a) The patient may have been lost in an unfamiliar place.

b) Colleagues detect poor work performance.

c) Those closest detect defects in the evocation of words and names.

d) When reading a paragraph from a book retains very little.

e) He can display a greatly diminished capacity in the memory of the new people he has known.

f) He may lost or placed in the wrong place a value object.

g) In the clinical examination he may show an apparent defect concentration.

A defect memory target is observed only with an intensive interview.

a decrease in yields appears in demanding work or social situations.

Denial or ignorance of the defect becomes manifest in the patient.

The symptoms are accompanied by discrete-moderate anxiety.

**GDS-4, moderate cognitive defect**

(Minimental cognitive scale of Lobo between 16 and 23 points). It corresponds with mild dementia:

clearly defined defects on careful clinical interview in the following areas:

a) decreased knowledge of current and recent events.

b) The patient may have a deficit in memory of his own personal history.

c) Default concentration shown in the serial subtraction.

d) decreased ability to travel, finances, etc.

Often there are no defects in the following areas:

a) orientation to time and person.

b) Recognition of people and familiar faces.

c) Ability to travel to familiar places.

Inability to perform complex tasks.

Denial is the dominant mechanism of defense.

Decreased affection and abandonment in the most demanding situations.

**GDS-5 moderate to severe cognitive defect**

(Minimental cognitive scale of Lobo between 10 and 19 points). It corresponds to a moderate stage dementia:

The patient can not survive long without some assistance.

Not remember relevant details of his current life: His address or phone, the names of close relatives (such as grandchildren), the name of the school, etc.

Often some disorientation to time (date, day of the week, season, etc.) or space.

A person with formal education may have difficulty counting backwards from 40 groups of four, or from 20 twos.

Maintains knowledge of many of the most interesting facts concerning himself and others.

Invariably knows his name, and usually his wife and children.

It does not require assistance in the toilet or food, but may have some difficulty in choosing the right dresses

**GDS-6, severe cognitive defect**

(Minimental cognitive scale of Lobo between 0 and 12 points). It corresponds to moderately severe dementia in the stadium:

He may occasionally forgets the name of the wife, which, moreover, is entirely dependent for survival.

Unknown events and recent experiences of his life.

It maintains some knowledge of his past life, but very fragmentary.

Generally unaware of their surroundings, year, season, etc.

It may be unable to count for 10 backward and sometimes forward.

It requires some assistance in daily activities.

He may has incontinence or require assistance to move, but he can goes to familiar places.

Diurnal rhythm is frequently altered.

Almost always remember his name.

Often still able to distinguish between familiar and unfamiliar persons in their emotional environment.and personality changes quite variable, such as:

a) delusional behavior: impostor can accuse his wife, or talking to nonexistent people, or its mirror image.

b) obsessional symptoms, as repeated cleaning activities.

c) Symptoms of anxiety, agitation and even violent behavior, previously inexistent.

d) cognitive Abulia, loss of desire, lack of development of a thought to determine a course of action purposing.

**GDS-7, very serious cognitive defect**

(Minimental cognitive scale of Lobo = 0 points, impracticable). It corresponds to a severe stage dementia:

progressive loss of verbal skills. Initially you can verbalize words and phrases very circumscribed; in the latter stages there is no language, only grunts.

Urinary incontinence. He requires assistance in grooming and feeding.

They are lost of basic psychomotor skills, such as ambulation.

The brain is unable to tell the body what to do. Appear frequently generalized and cortical signs and neurological symptoms.

Disclaimer: The correlation between stage and GDS score is indicative Minimental cognitive scale of Lobo

**ANNEX 5. Informed Consent Sheet**

|  |  | | | |  | |  | |  |  | | |  |  | |
| --- | --- | --- | --- | --- | --- | --- | --- | --- | --- | --- | --- | --- | --- | --- | --- |
|  | | |  | | |  | |  | |  | |  | |  |  |
|  | |  | | | | | |  | | | | | |  | |
| \|  \| \| --- \|   The patient ……………………………………………………………………………………………………………………………… ...  (Full name of patient)  -I read the information sheet that has given me  -I've been able to ask questions about the study  -I have received enough information about the study  I have spoken with …………………………………………………………………….  I understand that participation is voluntary.  I understand that I can withdraw from the study:  -whenever I want  without having to explain  without impacting on medical care or welfare or any other  Therefore I give my agreement freely to participate in the study.  Patient Signature Investigator Signature  ..../..../.... . .... / .... / .... ..../..../.... . .... / .... / .... | |  | | | | | |  | | | | | |  | |
|  | | | |  | | | | | | |  | | | | |
|  | |  | | | | | |  | | | | | |  | |
| **ANNEX 6. Patient information Sheet** | |  | | | | | |  | | | | | |  | |
| \| **Patient information sheet**  You has been invited to participate in a study on the development of mild cognitive impairment. Before you agree to participate in this study please read this page and feel free to ask any questions you have.  **WHAT IS THE PURPOSE OF THIS STUDY?**  Assess whether adding to the usual diet a food supplement marketed in Spain in capsules based on Omega-3 can be beneficial for symptoms of mild cognitive impairment (memory ...). Thus, two groups will be created; one of them will be provided with the product and the other not. All will perform the usual test performed in the residence. Some medical history data will be collected.  **DO I HAVE TO PARTICIPATE?**  Your participation in this study is completely voluntary.  **WHAT ARE THE DISADVANTAGES AND RISKS TO PARTICIPATE?**  It will not change the care or treatment that you are actually receiving.  **WILL IT BE CONFIDENTIAL MY PARTICIPATION IN THIS STUDY?**  The data collected will be treated with strict confidentiality in accordance with legal requirements on the treatment of personal data (Organic Law 15/1999 of December 13, "Protection of Personal Data").  **WHAT YOU WILL DO WITH THE RESULTS OF THE STUDY?**  These results (anonymous) will be disseminated in scientific journals forums and always maintaining the confidentiality of all participating patients.  Thanks for your attention. If you agree to participate in this study, you will be given a copy of this information sheet for the patient and a signed copy of the consent form. \|  \|  \|  \| \| --- \| --- \| --- \| --- \| | | | |  | | | | | | |  | | | | |

|  |  |  |
| --- | --- | --- |
